# Modulation of hepatic stellate cells by Mutaflor^®^ probiotic in non-alcoholic fatty liver disease management

**DOI:** 10.1186/s12967-022-03543-z

**Published:** 2022-07-30

**Authors:** Noha M. Hany, Sanaa Eissa, Manal Basyouni, Amany H. Hasanin, Yasmin M. Aboul-Ela, Nagwa M. Abo Elmagd, Iman F. Montasser, Mahmoud A. Ali, Paul J. Skipp, Marwa Matboli

**Affiliations:** 1 Department of Medical Biochemistry and Molecular Biology, Faculty of Medicine, Ain Shams University, Abbassia, P.O. box, Cairo, 11381 Egypt; 2 Department of Clinical Pharmacology, Faculty of Medicine, Ain Shams University, Cairo, Egypt; 3 Department of Medical Microbiology and Immunology, Faculty of Medicine, Ain Shams University, Cairo, Egypt; 4 Department of Gastroenterology, Hepatology and Infectious Diseases, Tropical Medicine, Faculty of Medicine, Ain Shams University, Cairo, Egypt; 5 Department of Molecular Microbiology, Military Medical Academy, Cairo, Egypt; 6 Centre for Proteomic Research, School of Biological Sciences, Faculty of Environmental and Life Sciences, University of Southampton, Southampton, UK; 7 MASRI Research Institue, Ain Shams University, Cairo, Egypt

**Keywords:** NAFLD, NASH, Probiotic, *E. coli*, Hepatic Stellate cells, Liver fibrosis, Hedgehog, Hippo

## Abstract

**Background:**

NAFLD and NASH are emerging as primary causes of chronic liver disease, indicating a need for an effective treatment. Mutaflor® probiotic, a microbial treatment of interest, was effective in sustaining remission in ulcerative colitis patients.

**Objective:**

To construct a genetic-epigenetic network linked to HSC signaling as a modulator of NAFLD/NASH pathogenesis, then assess the effects of Mutaflor^®^ on this network.

**Methods:**

First, in silico analysis was used to construct a genetic-epigenetic network linked to HSC signaling. Second, an investigation using rats, including HFHSD induced NASH and Mutaflor^®^ treated animals, was designed. Experimental procedures included biochemical and histopathologic analysis of rat blood and liver samples. At the molecular level, the expression of genetic (FOXA2, TEAD2, and LATS2 mRNAs) and epigenetic (miR-650, RPARP AS-1 LncRNA) network was measured by real-time PCR. PCR results were validated with immunohistochemistry (α-SMA and LATS2). Target effector proteins, IL-6 and TGF-β, were estimated by ELISA.

**Results:**

Mutaflor^®^ administration minimized biochemical and histopathologic alterations caused by NAFLD/NASH. HSC activation and expression of profibrogenic IL-6 and TGF-β effector proteins were reduced via inhibition of hedgehog and hippo pathways. Pathways may have been inhibited through upregulation of RPARP AS-1 LncRNA which in turn downregulated the expression of miR-650, FOXA2 mRNA and TEAD2 mRNA and upregulated LATS2 mRNA expression.

**Conclusion:**

Mutaflor^®^ may slow the progression of NAFLD/NASH by modulating a genetic-epigenetic network linked to HSC signaling. The probiotic may be a useful modality for the prevention and treatment of NAFLD/NASH.

**Supplementary Information:**

The online version contains supplementary material available at 10.1186/s12967-022-03543-z.

## Background

Non-alcoholic fatty liver disease (NAFLD) and its aggressive form, non-alcoholic steatohepatitis (NASH), are a financial burden on healthcare systems [1]. NAFLD/NASH can silently [2] progress to cirrhosis, hepatic failure, and hepatocellular carcinoma (HCC) [3]. Despite increasing global prevalence, currently at 25%, and grave complications [1], NAFLD/NASH had no satisfactory medical treatment until recently [4].

NASH leads to a state of progressive inflammation, then regeneration, and eventually fibrosis [5, 6]. Although many cell types have been implicated in this state but hepatic stellate cells (HSCs) have been explicit [7]. They are key players in hepatic inflammation, regeneration and their role in fibrosis is unequivocal [8]. HSCs are perisinusoidal progenitor cells [9] that can transform into fibrogenic cells [10]. Activation of HSCs is considered the core of liver fibrosis not only in NAFLD but in other chronic liver diseases, while the elimination of activated HSCs is pivotal for fibrosis resolution [8]. Multiple signaling pathways are implicated; however, the full pathogenesis underlying HSC activation and elimination in the liver are still unknown [11–15]. A major pathway, hedgehog (Hh) signaling, stimulates and regulates responses of HSCs [16]. Hh targets many transcription factors, including forkhead box A2 (FOXA2), an initiator of liver development during embryogenesis [17]. The pathway is activated directly by TEA domain transcription factor 2 (TEAD2) that increases expression of pro-fibrinogenic factors in HSCs [18]. Hippo signalling and its core component, large tumor suppressor kinase 2 (LATS2), another emerging HSC pathway [19]. Additionally, transforming growth factor β (TGF-β) [20] and interleukin-6 (IL-6) signaling are considered core fibrogenic HSC activators [21].

Epigenetics encompasses connections among multiple signaling pathways that affect HSCs [22]. Epimutations are indispensable for explaining pathogenesis of NASH [23]. microRNAs (miRs) and long noncoding RNAs (LncRNAs), two major epigenetic families [24], have pivotal roles in the reversible fine-tuning of signaling pathways [25], making them attractive biomarkers for the monitoring of disease progression and the effect of treatment [26].

Therapeutic use of probiotics for NAFLD in clinical trials has increased [27–29]. Escherichia coli Nissle (EcN), a nonpathogenic Gram-negative strain and the active component in Mutaflor^®^ (Ardeypharm GmbH, Herdecke, Germany and EcN, Cadigroup, In Italy) is used to treat gastrointestinal diseases [30–32]. EcN is a mesalazine alternative for maintaining ulcerative colitis remission [33, 34].

We were motivated to study the therapeutic effect of Mutaflor^®^ on HSCs in NAFLD/NASH. Initially, we used in silico data to assess hedgehog and hippo connected genetic players involved in the activation of HSCs (TEAD2, FOXA2, LATS2), there epigenetic modifiers (miR-650 and RPARP AS-1 LncRNA) and HSCs effecter proteins (IL-6 and TGF-β).We then compared the impact of Mutaflor^®^ on expression of these factors in sera and tissue of NASH model rats and healthy controls.

## Methods

**Fig. 1:**
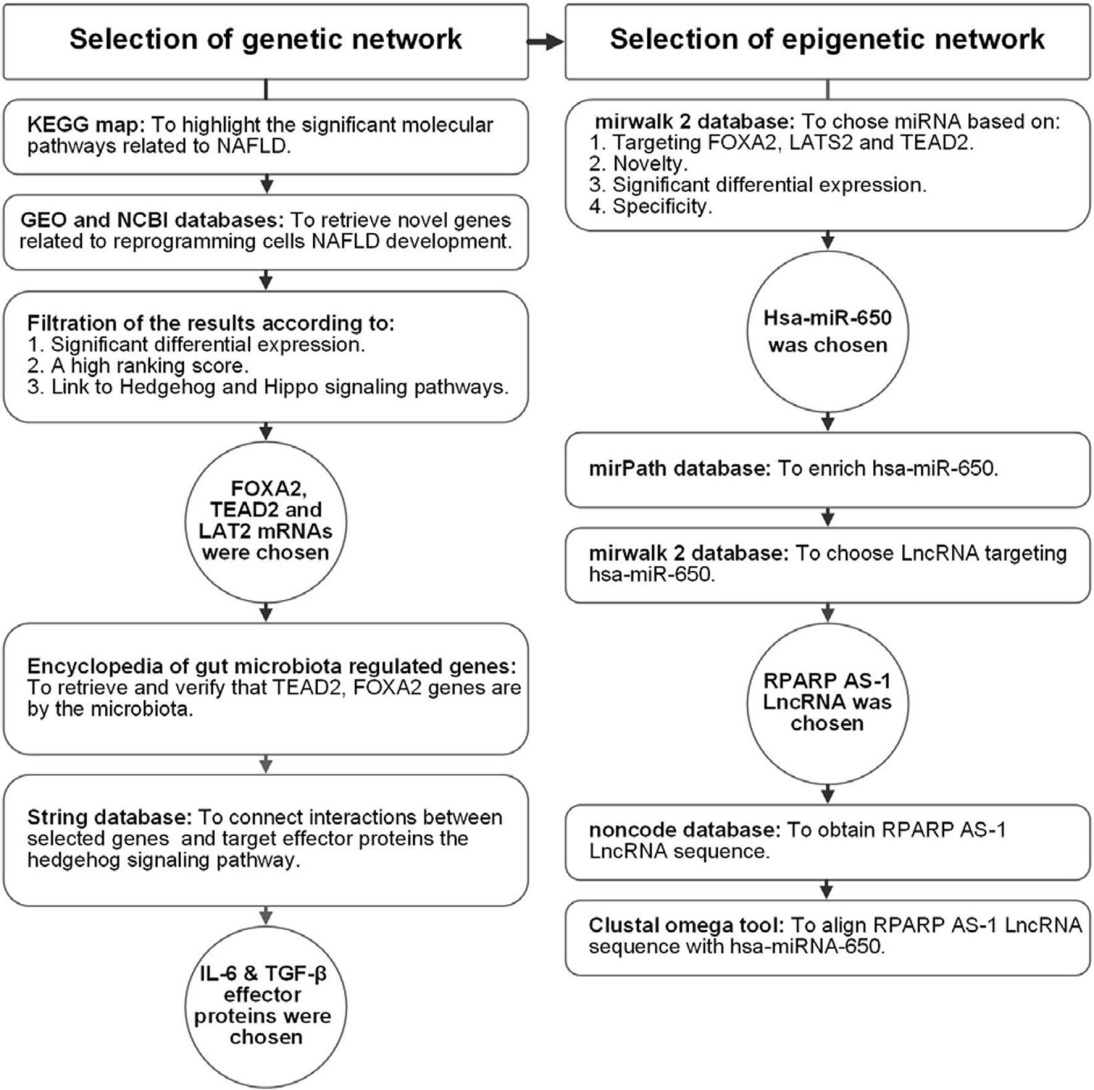
Workflow of bioinformatics steps

**Fig. 2:**
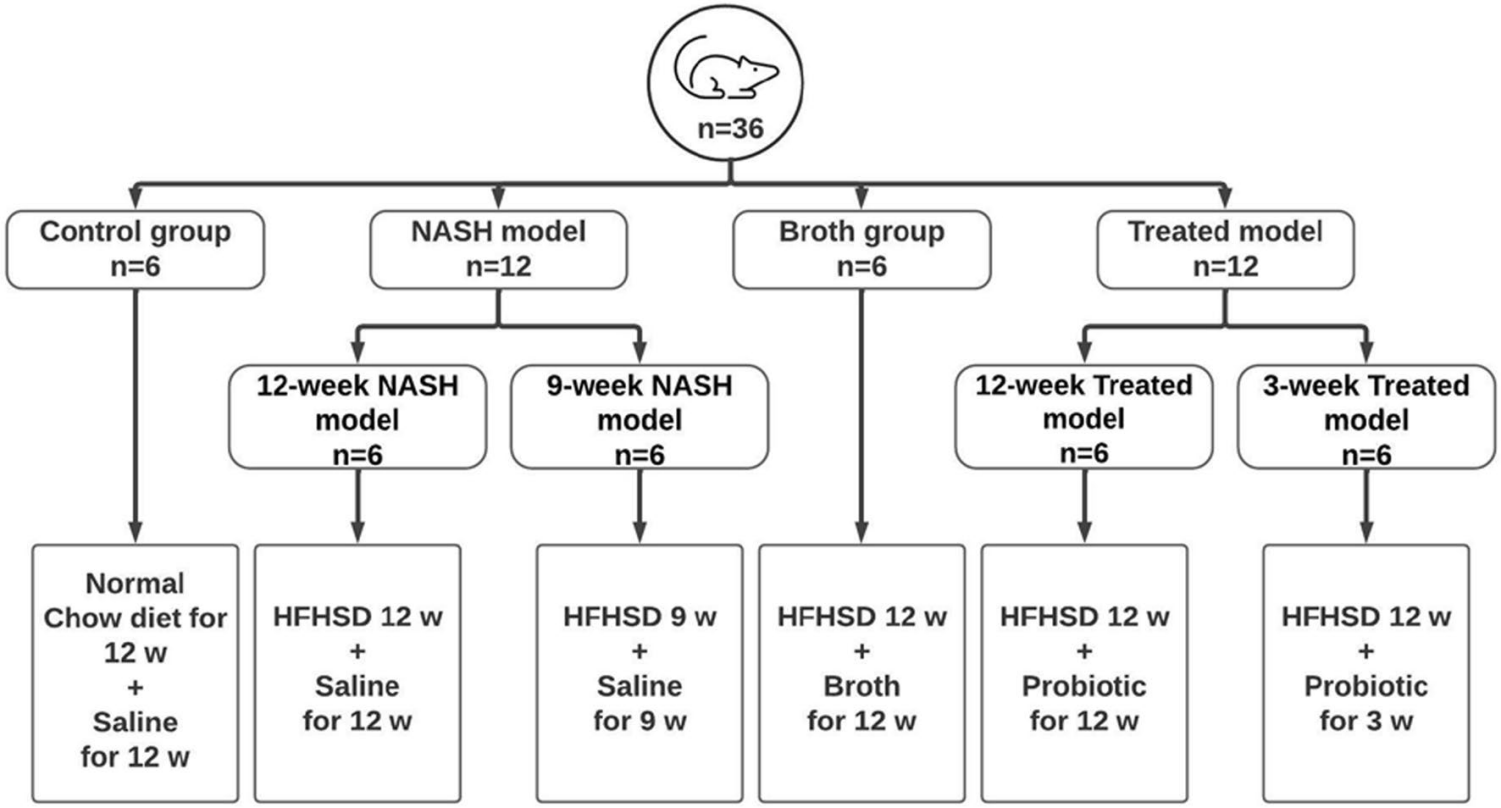
Workflow chart representing the experimental design of the animal groups. *NASH* nonalcoholic steatohepatitis, *HFHSD* high-fat high sucrose diet

**Fig. 3:**
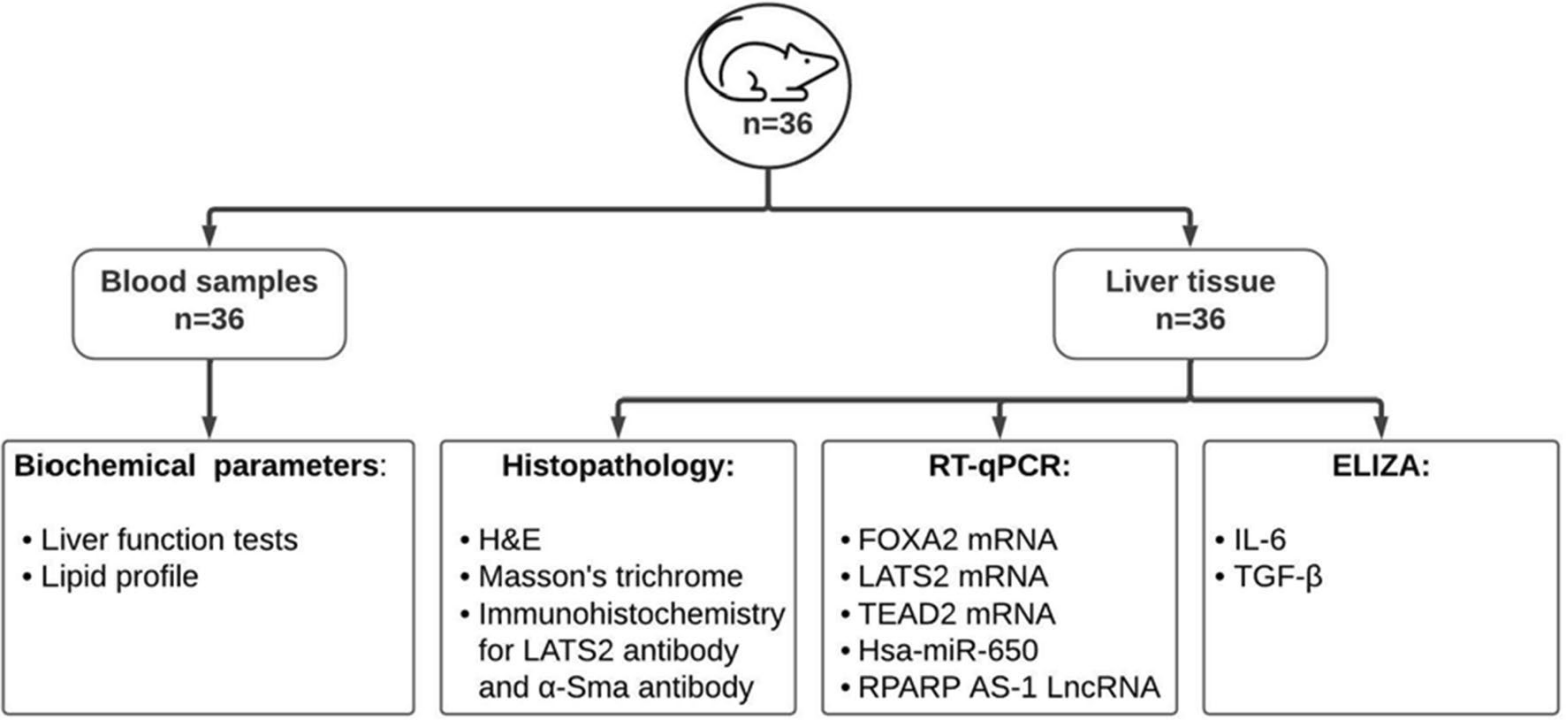
Experimental procedures carried out on rat blood and tissue samples to validate Insilco hypothesis

Initially, we used in silico data analysis to identify markers activating HSCs in NASH by assessing hedgehog and hippo connected genetic players (TEAD2, FOXA2, LATS2 mRNAs). alpha-smooth muscle actin (α-SMA) was assed as a marker of activated HSCs. IL-6 and TGF-β were retrieved as active HSC effector proteins Then, we retrieved epigenetic modifiers (miR-650 and RPARP AS-1 LncRNA) (Figs. 1, 2 and 3). Finally, we validated these network and compared the effects of Mutaflor^®^ on their expression in sera and tissues of NASH-induced animals and controls.

### Bioinformatic-based selection of genetic and epigenetic networks

Selection of the genetic network (Fig. 1): Initially, we used the KEGG map (available at https://www.genome.jp/kegg-bin/show_pathway?hsa04932) to highlight significant molecular pathways related to NAFLD (Additional file 1: Fig. S1A, B). Next, we used GEO and NCBI databases to retrieve novel genes related to cell reprogramming and NAFLD development. Filtering results identified genes FOXA2, TEAD2 and LATS2 with significant differential expression, a high-ranking score, and links to hedgehog and hippo signaling IDs (51565912, 10634719 and 106305647, respectively) (Additional file 1: Fig. S2A–C). Secondary verification of chosen genes used the encyclopedia of gut microbiota regulated genes (available at http://microbiota.wall.gu.se/) to retrieve specific genes (TEAD2, FOXA2) regulated by gut microbiota as a potential step toward targeting these microbiota (Additional file 1: Fig. S3A-B). String (available at https://string-db.org/) was used to connect interactions among selected genes (TEAD2, FOXA2, LATS2), target effector proteins (IL-6 and TGF-β), and the hedgehog signaling pathway (Additional file 1: Fig. S4).

Selection of epigenetic regulators: hsa-miR-650 was chosen from the mirwalk 2 database based on novelty, significant differential expression, and specificity to the liver (available at http://mirwalk.umm.uni-heidelberg.de/interactions/?mirnaid=hsa-miR650&genesymbol=&bindingp=0.95&position=3UTR&targetscan=0&mirdb=0&mirtarbase=0&submit=set+filter). The mirPath database was used to enrich hsa-miR-650 (available at http://snf-515788.vm.okeanos.gr/#mirnas=hsa-miR-650; hsa-miR-650 & methods = TargetScan; Tarbase & selection = 0), which revealed that miR-650 is linked to Hh, NAFLD, and MAPK signaling (Additional file 1: Fig. S5), and targets FOXA2, LATS2 and TEAD2 (Additional file 1: Fig. S6 A-C). Finally, RPARP AS-1 LncRNA was chosen first by choosing LncRNA targeting miR-650, using mirwalk2 (http://zmf.umm.uniheidelberg.de/apps/zmf/mirwalk2/mir2ret/mir2mwlnc.php). We then aligned the LncRNA sequence obtained from noncode (available at http://www.noncode.org/) with miR-650 using the Clustal omega tool of the European Bioinformatics Institute (available at https://www.ebi.ac.uk/Tools/services/web/toolresult.ebi?jobId=clustalo-I20210209-113823-0177-82478586-p1m&analysis=alignments) (Additional file 1: Fig. S7A–D).

### Experimental animals (Fig. 2)

Thirty-six adult male Wistar rats (150–200 g) were purchased from the National Research Institute (Cairo, Egypt) and allowed one-week acclimatization. Rat chow was purchased from Meladco for Animal Food, El-Obour, Egypt. Pellets and tap water were available ad libitum unless indicated otherwise. Animals were bred and kept at 22 ± 2 °C, 55 ± 5% relative humidity, and a 12-h light/dark cycle (6:00 am–6:00 pm). Cages were cleaned daily. All effort was made to minimize animal suffering as well as reduce the number of animals. At the end of the experiment, rats were weighed, anaesthetized (1.2 g/kg urethane in distilled water; intraperitoneal) then euthanized. Blood samples were collected and processed, and liver specimens were weighed and processed.

All procedures were approved by and conducted following the Research Ethics Committee, Faculty of Medicine, Ain Shams University (FMASU-REC) requirements (FMASU MD 85/2020). FMASU-REC operates under Federal Wide Assurance No. 000017585.

#### Experimental groups

Animals were divided into four main groups. All treatments were administered orally by gavage. Group 1 (Control) rats were fed a standard chow diet and administered 1 mL of sterile saline daily for 12 weeks (n = 6). Group 2 (NASH model) rats were further subdivided into Group 2A (9-week NASH model) rats fed a high-fat high sucrose diet (HFHSD) and administered 1 mL of sterile saline daily for 9 weeks (n = 6); Group 2B (12-week NASH model) rats were fed HFHSD and administered 1 mL of sterile saline daily for 12 weeks (n = 6). Group 3 (Treated) rats were also subdivided into two groups. Rats in Group 3A (12-week Treated model) were fed HFHSD and administered probiotic (1 × 10^8^ colony-forming units (CFU)/mL) daily from day one for 12 weeks (n = 6); rats in Group 3B (3-week Treated model) were fed HFHSD for 12 weeks and administered probiotic daily for the last 3 weeks beginning at week 10 (n = 6). Group 4 (Broth) rats fed HFHSD for 12 weeks and administered 1 mL of Luria Bertani broth daily from day one for 12 weeks (n = 6).

#### Chemical, drug, and diet preparation

The probiotic Escherichia coli Nissle 1917 strain (Mutaflor^®^, Ardeypharm GmbH, Herdecke, Germany) was grown on Luria Bertani (LB) medium (HiMedia, Mumbai, India). The number of viable bacterial cells in one mL was determined using the plate counting method and calculated as Colony-forming units per mL (CFU/ mL) = no. of colonies × dilution factor [35]. A dose of 1 × 10^8^ CFU/mL was administered daily by gavage to treated animals [36–39]. Cholesterol and bile salts were purchased from Ralin BV (Lijinbaan, Netherlands) (Catalogue number 81254). LB broth was purchased from HiMedia (Mumbai, India, Catalogue No. M1245).

NASH was induced by feeding rats HFHSD consisting of 68.75% standard chow, 20% lard, 10% sucrose, 1% cholesterol, and 0.25% bile salts [40]. Components were mixed using water, made into pellets and left to dry. The diet was prepared every five days and stored in the refrigerator.

#### Experimental procedures

Procedures are summarized in Fig. 3.

##### Processing of blood samples

Rats were fasted for 12 h at the end of the study then anesthetized with a single dose of urethane (1.2 g/kg, IP) before sacrifice. Blood samples were collected from retro-orbital veins and centrifuged at 3000 rpm for 10 min for serum separation. Aliquots of sera were stored at − 20 °C for subsequent biochemical analysis.

##### Biochemical parameters

Serum aspartate transaminase (AST), alanine transaminase (ALT), gamma-glutamyl transferase (GGT), alkaline phosphatase (ALP), albumin, total bilirubin, direct bilirubin (TB and DB, respectively), triglycerides (TGs), total cholesterol (Tc), low-density lipoprotein (LDL) and high-density lipoprotein (HDL) cholesterol were assessed in sera by commercial kits using an automated Beckman Coulter AU680 autoanalyzer (Beckman Coulter Inc, CA).

##### Histological examination

Whole livers were removed promptly, weighed, and dissected. Hepatic tissue samples were immediately stored at − 80 °C for RNA and protein assessment. Remaining tissues were rapidly fixed in 10% neutral buffered formalin for histopathological and immunohistochemical analyses.

#### Quantitative expression of RNA using RT-qPCR

Extraction of total RNA, lncRNA, and miRNA: miRNeasy Mini Kit (Cat. No. 217004, Qiagen) was used to extract total RNA from tissue samples following the manufacturer’s protocol. Nanodrop from Thermo Scientific (USA) was used to assess the integrity and concentration of RNA; purity of isolated RNA was 1.8–2.

Reverse transcription of cDNA: Total RNA was immediately reverse transcribed into complementary DNA (cDNA) using an RT2 First-Strand kit (Cat. Nos. 303404, Qiagen) for both mRNAs and LncRNA and a mi Script II RT Kit (Cat. Nos. 218160, 218161, Qiagen) for miRs. A Thermo Hybaid PCR Express Thermal Cycler (Thermo Fisher, Massachusetts, USA) was used following the manufacturer’s protocol.

mRNA/miR/LncRNA network quantitative expression using RT-qPCR: A QuantiTect SYBR^®^ Green PCR Kit (Cat. No. 204143, Qiagen, Germany) and a QuantiTect Primer Assay (NM 021784, NM 003598, and NM 014572) were used to detect FOXA2, TEAD2, and LATS2 mRNAs expression in liver tissue samples. An RT2 SYBR Green Rox qPCR Master Mix (Cat. No. 330500, Qiagen, Germany) and RT2 LncRNA qPCR Assay (ENST00000473970, Catalog No. 33070, Qiagen, Germany) were used to quantify RPARP AS-1 LncRNA in liver tissue. A miScript SYBR Green PCR Kit (Cat. No. 218073, Qiagen, Germany) and miR650 miScript Primer Assay targeting mature miR:miR650 (MIMAT0003320, Cat. No. 218300, Qiagen, Germany) were used to assess miR-650 expression in tissue samples, following the manufacturer’s procedure. GAPDH and SNORD72 were used as housekeeping genes to standardize raw data before comparing them to reference. SYBR green-based qPCR software was used in an Applied Biosystems 7500 FAST Real-Time PCR machine to perform qPCR (Applied Biosystems, USA). Reaction conditions were denaturation at 95 °C for 15 min, followed by 40 cycles of denaturation at 94 °C for 10 s and annealing at 55 °C for 30 s. Final extension was at 70 °C for 30 s. Each reaction was performed in duplicate. Applied Biosystems Stepone plusTM Software v2.2.2 was used to calculate threshold cycles (Ct) for each sample. Ct greater than 36 is considered negative. RQ of RNA expression was calculated using the Livak technique, where RQ = 2^−ΔΔCt^. Melting curves were examined to confirm amplicon specificities for SYBR Green-based PCR amplification.

#### Immunohistochemistry

Paraffin tissue sections were deparaffinized with xylene and hydrated using a series of decreasing alcohol concentrations. The activity of endogenous peroxidase was blocked by incubation in 3% hydrogen peroxide (H_2_O_2_) solution in methanol for 10 min. H_2_O_2_ was washed away by rinsing with phosphate-buffered saline (PBS). A set of tissue sections was incubated overnight at 4 °C with diluted 1:400 mouse monoclonal anti-SMA antibodies (Sigma Aldrich^®^, catalog A2547-0.5ML, USA). Another set of tissue sections was incubated for two hours at room temperature with 1:400 dilution of anti-LATS2 antibody (abcam^®^, catalog ab111054L, UK) then rinsed with PBS. Tissue sections were then incubated with biotinylated secondary antibodies for 30 min, then in diaminobenzidine (DAB) solution (Vector Laboratories, Inc., Burlingame, CA, USA) to reactivate peroxidase. Counterstaining with hematoxylin was followed by rinsing in tap water for 10 min. Finally, tissue was dehydrated and examined for color changes. Cells positive for α-SMA and LATS2 antibody staining were identified by dark brown nuclei.

Histoscore (H-score) was calculated as described by Khatun et al. [41] [H-score = (% of stained cells) × (intensity of staining grade + 1)], where cell staining intensity was scored as 0 for no staining, 1 for weak staining, 2 for moderate, and 3 for strong. The percentage of positive cells for α-SMA and LATS2 were counted as fractional area by Image J software.

#### Estimation IL-6 and TGF-β protein levels in liver tissue

A quantitative investigation of IL-6 and TGF-β protein expression in tissue samples used Rat IL-6 Kit (Catalog No: E0079r, EIAAB SCIENCE INC, WUHAN) and Rat TGF-β ELISA Kits (Catalog No: E0124r, EIAAB SCIENCE INC, WUHAN), respectively, following the manufacturer’s protocols.

#### Statistical analysis

Data are expressed as mean ± SD. Kolmogorov–Smirnov and Shapiro–Wilk tests were used to assess normality of data distributions. Data comparisons used GraphPad (version 8) for one-way ANOVA with post hoc Bonferroni's analysis. Box plots and IL-6 and TGF-β quantities were calculated. Correlation were assessed using SPSS software (version 20).

## Results

### Body and liver weight changes in NAFLD:

**Fig. 4:**
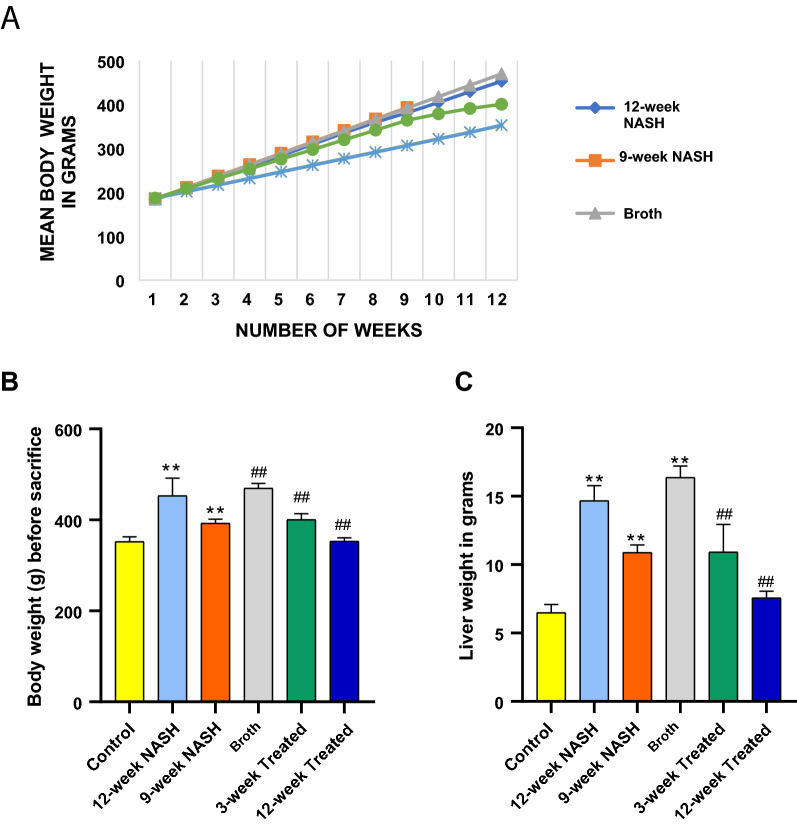
Effect of NASH induction and Probiotic administration on body weight and liver weight expressed in grams. **A** Mean body weight of each group for 12 weeks except 9-week NASH group. **B** Mean body weight of each groupat the end of the study, calculated in grams before sacrifaction at 12 weeks for all groups except for the 9-week NASH group, values are expressed as means ± SD. **C** Liver weight, values are expressed as means ± SD (n = 6). *p < 0.05 **p < 0.01 symbols are used when groups are compared with Control group while ^#^p < 0.05 and ^##^p < 0.01 symbols are used when groups are compared to 12-week NASH group

No significant differences in body weight among groups of rats were apparent at the beginning of the experiment (weight from 150 to 200 g, F = 0.126, *p* = 0.985). Body weight was measured every week for each rat individually. All animals showed a steady weight gain throughout the study. Significant differences in weight gain among groups of rats first appeared in the third week (F = 4.5*, p = 0.04) (Fig. 1). These differences persisted until the end of the study. Induction of NASH increased body weight gain; Broth rats had the largest body weight gain followed by 12-week, and 9-week NASH rats gained  256%, 246% and 213% of original body weight, respectively. Control rats showed only a 190% body weight gain. Final body weights of 12-NASH animals were 100.8 g more than controls, while 9-week NASH animals were 40.8 g more than controls (Fig. 4B).

Livers were weighed immediately after sacrifice. A significant difference in liver weight was seen between groups of rats (F = 56.2*, *p* < 0.01) (Fig. 4C). Induction of NASH showed an increase in liver weight with the largest mean liver weight reaching 16 g in Broth group rats. 12-NASH animals were 8.2 g more than controls, while 9-week NASH animals were 4.4 g more than controls (Fig. 4C). Final body weight was positively correlated with liver weight in Broth, 12- and 9-week NASH rats (r = 0.9*, p < 0.01).

### Biochemical parameters

#### Liver function

Serum levels of AST, ALT, GGT, ALP, TB, and DB were significantly different among the six groups of rats (F = 39.3*, 78.1*, 93.9*, 8.38* and 8*, respectively; and p < 0.001). These indicators of hepatocellular damage increased significantly with induction of NASH in 12- and 9-week NASH group animals when individually compared to controls (p < 0.001, except TB and DB, p = 0.01 and 0.004 respectively). Broth group rats showed no significant differences when compared with NASH group animals (p > 0.05). Induction of NASH disrupted normal lipid profiles. Compared to controls, 12-week NASH, 9-week NASH and Broth group rats displayed a significant increase in the serum levels of fasting TGs, total cholesterol, and LDL concomitant with a significant decrease in HDL levels (p < 0.001) (Table 1).

**Table 1:** The effect of NASH induction and probiotic administration on liver function tests and lipid profile

Groups	Control		12-week NASH		9-week NASH		Broth		3-week Treated		12-week Treated		F	p-value
	mean	SD	mean	SD	mean	SD	mean	SD	mean	SD	mean	SD		
SGOT	72.5	7	155.2	4.1	131.5	5.4	144.5	5.2	92.5	7.7	70.3	5.5	39	< 0.001**
SGPT	26.2	2.4	127.2	9.1	113.8	8.1	114.3	4.1	36.7	3.5	26.2	1.5	78	< 0.001**
GGT	13.8	1.8	66.5	3.8	72.8	3.6	71.8	4.5	25.0	1.1	17	1	94	< 0.001**
TB	0.4	0	1	0.2	1	0.2	1	0.1	0.4	0	0.4	0	8	< 0.001**
DB	0.1	0	0.3	0.1	0.3	0.1	0.3	0	0.2	0	0.1	0	8	< 0.001**
ALP	47.0	0.9	110.2	17.2	101.7	1.9	98.7	2.9	66.0	1.4	61.2	2.3	13	< 0.001**
T. Chole	144.9	2.5	253.6	11.3	228.5	8	246.6	12.7	137.8	4.1	139.5	3.9	49	< 0.001**
TG	52.7	2.9	182.2	9.7	169.2	7.5	166.3	13.4	92.5	5.2	75.7	4	49	< 0.001**
HDL	66.5	3	32.7	1.1	34.2	1.8	43.2	0.9	50.7	2.4	53.8	1.6	43	< 0.001**
LDL	67.8	2.5	184.5	9.4	160.5	7.3	170.2	11.9	68.7	2.2	70.5	3	64	< 0.001**

*F* one way anova

**p-value < 0.001 = highly significant difference

### Histopathological changes in NAFLD

Gross examination of freshly excised rat liver showed a marked difference between control rats compared with Broth and NASH group animals. Control livers had a shiny polished red appearance, while Broth and the NASH models showed a dull red appearance with whitish-yellow spots distributed across the liver surface.

**Fig. 5:**
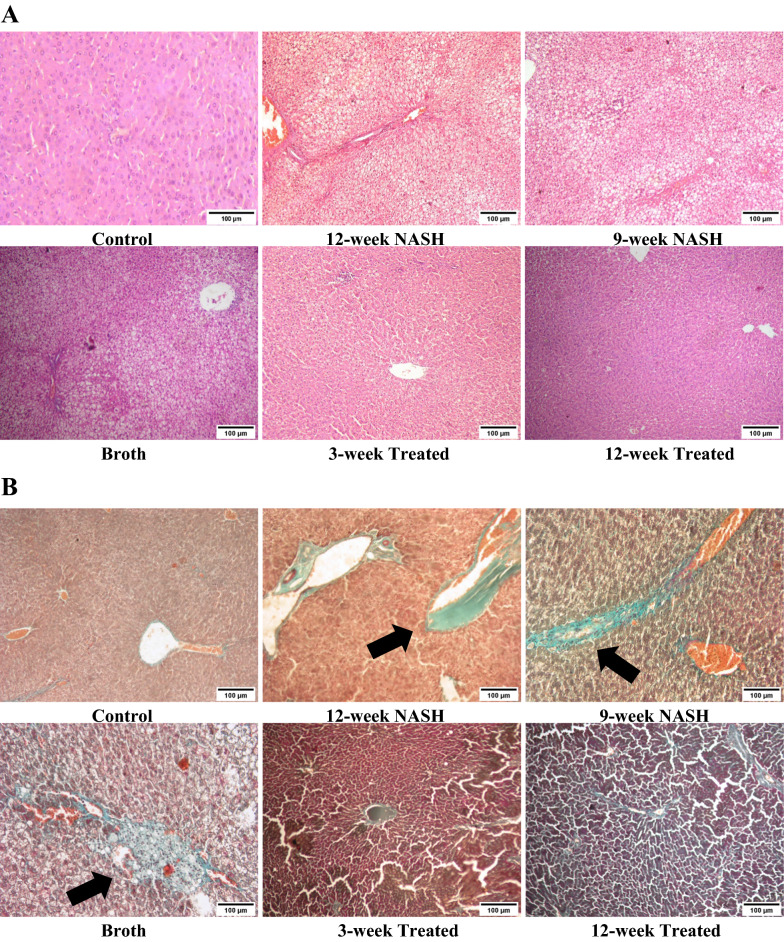
The effect of NASH induction and Probiotic use on steatosis, inflammation and fibrosis of the liver using HE (**A**) and Masson’s trichrome staining (**B**) (Magnifications: ×100). Liver histological examinations showed both macro- and microvesicular steatosis, lobular inflammation, focal necrosis, hepatocellular ballooning, and extensive fibrosis (Arrow) in NASH models. These effects were minimized with Probiotic were remarkable especially in 12-week Treated model

Histopathological grading of NAFLD was done according to Takahashi et al. [42]. Microscopically, hematoxylin and eosin (H&E) was used to assess the degree of steatosis and inflammation, and Masson’s trichrome stain was used to assess fibrosis. Control livers showed normal liver architecture with classical hexagonal hepatic lobules and no evidence of inflammation or fibrosis. Conversely, rats in Broth and NASH groups displayed a distortion of hepatic lobular architecture with evidence of macro- and micro-vesicular hepatic steatosis, lobular inflammation, cellular infiltration, ballooning of hepatocytes, and dilatation in central and the portal veins. Fibrosis was present along sinusoids around hepatocytes and in periportal areas (Table 2 and Fig. 5).

**Table 2:** The effect of NASH induction and probiotic administration on histopathological grading of the liver

Groups	Control	12-week NASH	9-week NASH	Broth	3-week Treated	12-week Treated
Steatosis	0/3	3/3	3/3	3/3	2/3	1/3
Ballooning	0/2	2/2	2/2	2/2	1/2	1/2
Inflammation grade	0/3	3/3	3/3	3/3	2/3	1/3
Fibrosis stage	0/4	4/4	3/4	4/4	2/4	1/4

### Immunohistochemical analysis for LATS2 and α-SMA in NAFLD

**Fig. 6:**
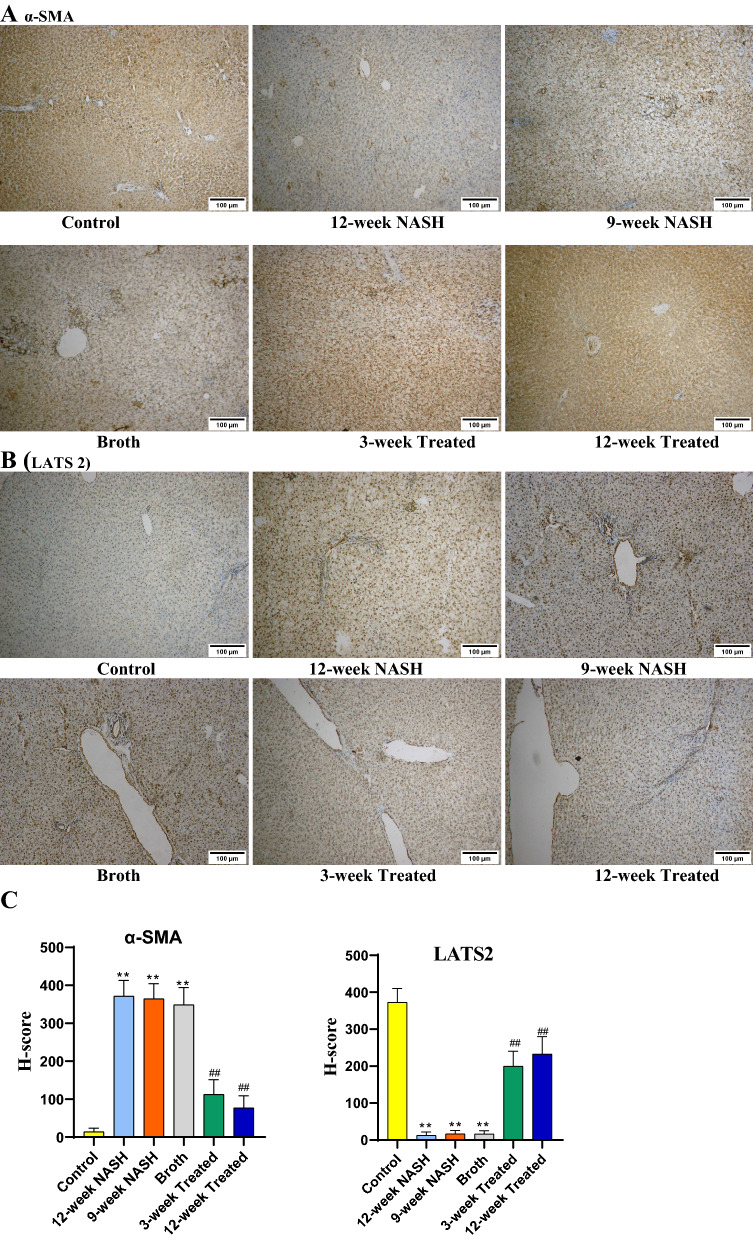
**A** Immunohistochemical staining (IHC) of α-SMA and **B** LATS 2 in liver sections from separate groups as indicated in the figures (Magnifications: ×100). **C** Statistical difference in α-SMA and LATS2 H-score among study groups. IHC assays demonstrated that liver tissue of NASH rats had more α-SMA-positive cells were localized in areas with inflammatory cells and areas with remarkable perisinusoidal fibrosis. The expression of α-SMA showed a significant increase in 12-week NASH group, 9-week NASH group and Broth group when compared with the Control group, However, in 12-week Treated and 9-week Treated groups, there was an evident decrease in the α-SMA expression. IHC assays of LATS2 were markedly decreased in 12-week NASH, 9-week NASH and Broth groups when compared to the Control group. However, 12-week Treated and 9-week Treated groups showed a significant increase in LATS2 expression when compared to the 12-week NASH group

Immunohistochemical analysis was used to assess the expression of α-SMA indicating the activity of HSCs and to assess and confirm LATS2 expression. Induction of NASH markedly increased α-SMA expression, indicating an increased number of activated HSCs. This effect was seen in NASH 12-week, NASH 9-week, and Broth group rats. Conversely, induction of NASH decreased LATS2 expression in NASH 12-week, NASH 9-week, and Broth group rats when compared individually with controls (Fig. 6).

### Altered expression of FOXA2, TEAD2, and LATS2 mRNAs, miR-650, and RPARP AS-1 LncRNA in NAFLD

**Fig. 7:**
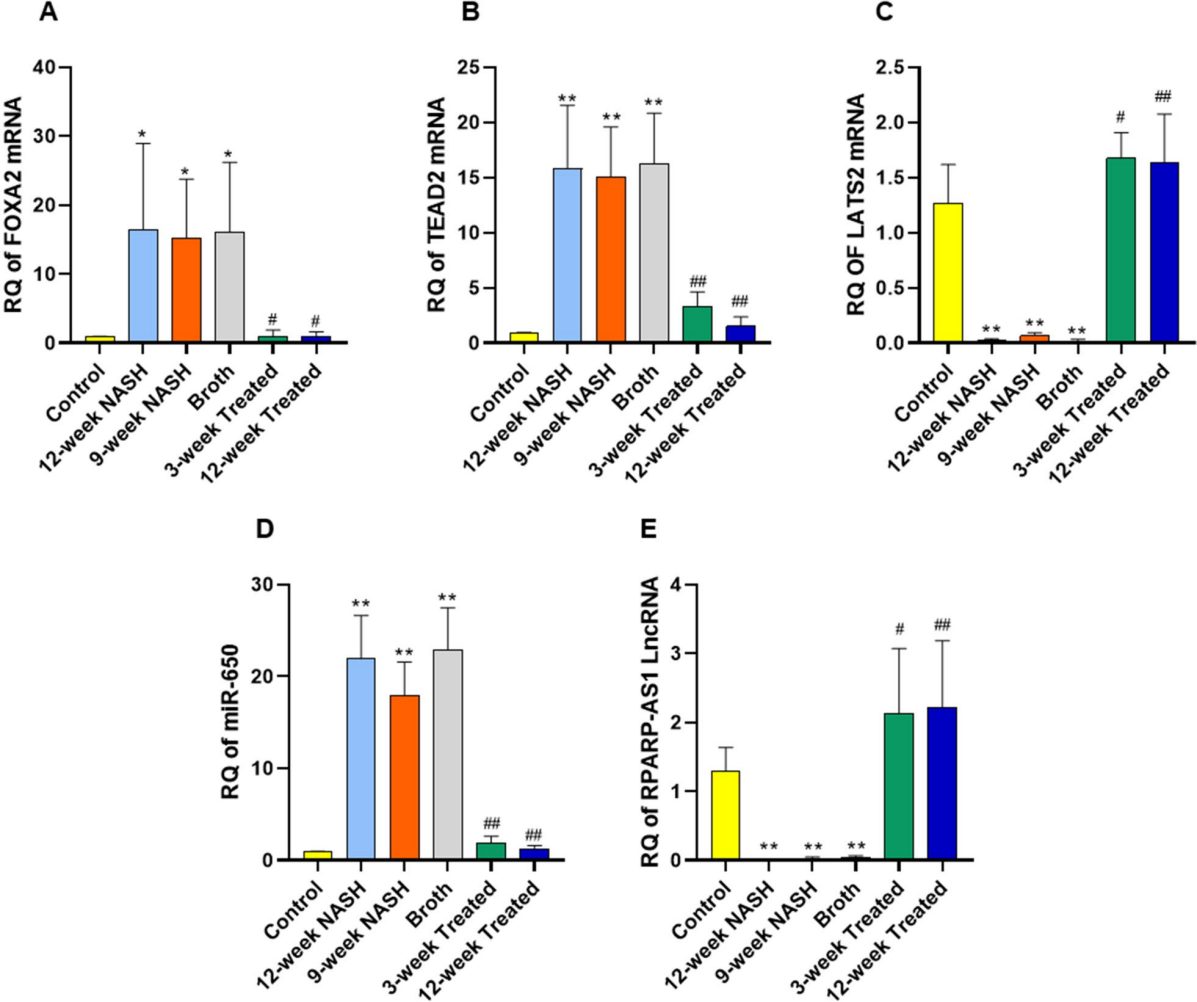
Effect of NASH induction and Probiotic administration on the expression level of hepatic FOXA2 mRNA, TEAD2 mRNA and LATS2 mRNA (**A**–**C**), hepatic miR-650 (**D**) and hepatic RPARP-AS1 LncRNA (**E**). Values are mean ± SD; number of animals = 6 rats/each group. *p < 0.05 **p < 0.01 symbols are used when groups are compared with Control group while ^#^p < 0.05 and ^##^p < 0.01 symbols are used when groups are compared to 12-week NASH group. RQ, relative quantification

Induction of NASH increased expression of FOXA2, and TEAD2 mRNAs, and miR-650 compared to controls. RQs of FOXA2 mRNA in 12-week NASH, 9-week NASH, and Broth groups rats individually with controls showed significant increases (16.5-, 15-, and 16-fold; *p*-values = 0.016, 0.035 and 0.02, respectively) (Fig. 7). Furthermore, RQs of TEAD2 mRNA in 12-week NASH, 9-week NASH, and Broth group animals with controls showed significant increases (16, 15, and 15-fold, respectively; *p* < 0.01). Similarly, RQ expression of miR-650 in 12-week NASH, 9-week NASH and Broth group animals with controls showed significant 22-, 18- and 23-fold increases, respectively; *p* < 0.01.

Conversely, induction of NASH significantly decreased RQs of LATS2 mRNA and RPARP AS-1 LncRNA; the RQ of LATS2 mRNA in 12-week NASH, 9-week NASH, and Broth group animals individually with controls showed a significant decrease (45, 14, and 50-fold, respectively; *p* < 0.01). Also, RQ of RPARP AS-1 LncRNA in the same groups of rats showed significant 67-, 31-, and 23-fold decreases, respectively; *p* < 0.01.

### Altered relative concentrations of target effector proteins, IL-6 and TGF-β in NAFLD

**Fig. 8:**
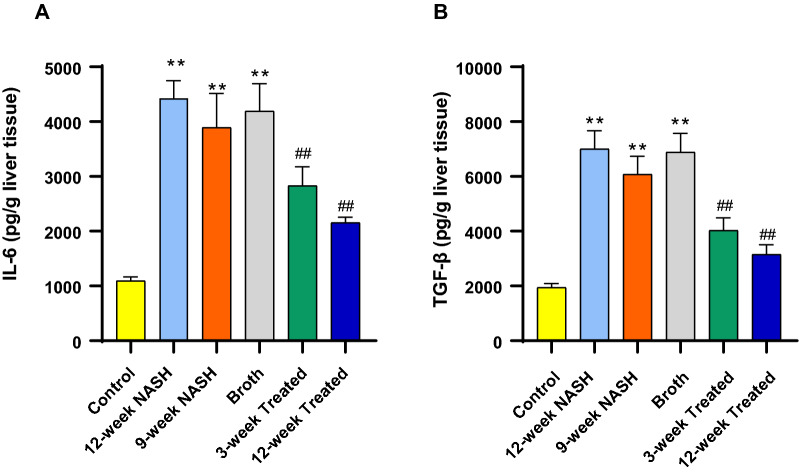
Effect of NASH induction and Probiotic administration on **A** Relative concentration of IL-6 and **B** TGF-β/g liver tissue. Values are mean ± SD; number of animals = 6 rats/each group. *p < 0.05 **p < 0.01 symbols are used when groups are compared with Control group while ^#^p < 0.05 and ^##^p < 0.01 symbols are used when groups are compared to 12-week NASH group. *(pg/g)* picogram per gram liver tissue

Induction of NASH significantly increased the level of IL-6 and TGF-β when compared with normal controls. For example, 12-week NASH group animals showed approximately 4- and 3.6-fold increases in IL6 and TGF-β concentrations, respectively (p < 0.01); similar 3.6- and 3-fold increases were seen in IL6 and TGF-β concentration in the 9-week NASH rats, respectively (p < 0.01). Also, Broth group animals showed 3.8- and 3.5-fold increases in IL-6 and TGF-β concentration, respectively (p < 0.01) (Fig. 8).

### Correlations among quantitative expression levels of mRNA, miRNA, LncRNA, target effector proteins and HSCs activation

FOXA2 mRNA was significantly correlated with TEAD2 mRNA and miR-650 (r = 0.77, 0.74, *p* < 0.01). A significant negative correlation was observed with LATS2 mRNA and RPARP AS-1 LncRNA (r = − 0.75 and − 0.74, respectively, *p* < 0.01). Similarly, IL6 and TGF-β concentration were significantly and positively correlated with FOXA2, TEAD2 mRNAs, and miR-650 expressions (*p* < 0.01) in all groups of animals. Concentrations and expressions were significantly and negatively correlated with LATS2 mRNA and RPARP-AS1 LncRNA (*p* < 0.01). HSCs activation, indicated by increase in α-SMA H-score, was significantly correlated with increase in FOXA2, TEAD2 mRNAs, and miR-650 expressions (r = 0.7, 0.9 and 0.7, respectively, *p* < 0.01). and with decease in LATS2 mRNA and RPARP-AS1 LncRNA expression (r = − 0.8and − 0.75, respectively, *p* < 0.01) (Table 3).

**Table 3:** Spearman’s correlation between the RQ of the selected mRNAs, miRNA, LncRNA, effector protein concentration and HSCs activation

Biomarker		FOXA2 mRNA	TEAD2 mRNA	LATS2 mRNA	miR-650	RPARP-AS1 LncRNA	IL6	TGF-β	α-SMA H-score
FOXA2 mRNA	r	1	0.77	− 0.75	0.74	− 0.74	0.75	0.75	o.74
	Sig		< 0.01^a^	< 0.01^a^	< 0.01^a^	< 0.01^a^	< 0.01^a^	< 0.01^a^	< 0.01^a^
TEAD2 mRNA	r	0.77	1	− 0.67	0.84	− 0.64	0.89	0.87	0.85
	Sig	< 0.01^a^		< 0.01^a^	< 0.01^a^	< 0.01^a^	< 0.01^a^	< 0.01^a^	< 0.01^a^
LATS2 mRNA	r	− 0.75	− 0.67	1	− 0.72	0.82	− 0.7	− 0.74	− 0.71
	Sig	< 0.01^a^	< 0.01^a^		< 0.01^a^	< 0.01^a^	< 0.01^a^	< 0.01^a^	< 0.01^a^
miR-650	r	0.74	0.84	− 0.72	1	− 0.74	0.85	0.9	0.79
	Sig	< 0.01^a^	< 0.01^a^	< 0.01^a^		< 0.01^a^	< 0.01^a^	< 0.01^a^	< 0.01^a^
RPARP-AS1 LncRNA	r	− 0.75	− 0.64	0.82	− 0.74	1	− 0.71	− 0.74	− 0.74
	Sig	< 0.01^a^	< 0.01^a^	< 0.01^a^	< 0.01^a^		< 0.01^a^	< 0.01^a^	< 0.01^a^
IL6	r	0.75	0.89	− 0.7	0.85	− 0.71	1	0.95	0.84
	Sig	< 0.01^a^	< 0.01^a^	< 0.01^a^	< 0.01^a^	< 0.01^a^		< 0.01^a^	< 0.01^a^
TGF-β	r	0.75	0.87	− 0.74	0.9	− 0.74	0.95	1	0.8
	Sig	< 0.01^a^	< 0.01^a^	< 0.01^a^	< 0.01^a^	< 0.01^a^	< 0.01^a^		< 0.01^a^

*r* Spearman’s correlation coefficient

^a^Sig < 0.001 = highly significant correlation

### Probiotic prevented the increase in body and liver weights

Probiotics administration for 12 weeks normalized NASH effects on body weight gain. Probiotic lowered average weight gain from 24.5 g/week in 12-week NASH to 15 g/week in 12-week Treated rats. The significant probiotic effect was first observed in the third week between 12-week treated and 9-week NASH rats (mean difference = − 20.3* g, p-value = 0.03). Although, it appeared in the fourth week between 12-week treated and 12-week NASH rats (mean difference = − 27* g, p-value < 0.01), this effect was consistent throughout the experiment. 12-week treated rats showed a 189.3% body weight gain significantly less than 12-week NASH rats (mean difference = − 100* g, p-value < 0.01). Final Body weight showed no significant difference between 12-week Treated rats and Controls (p-value = 1). Probiotic administration for 3 weeks also decreased NASH effects on body weight gain. 3-week Treated rats showed a steady weight gain until the last 3 weeks probiotic lowered average weight gain from 22 to 14.8 g/week. 3-week (Fig. 4A). Treated rats showed a 215% body weight gain which was significantly less than 12-week NASH animals (mean difference = − 52.5* g, p-value < 0.01), although, still significantly higher than control groups (mean difference = 48.5*g, p-value < 0.01) (Fig. 4B).

Liver weight was significantly decreased in both 3-week and 12-week Treated animals when compared to 12-week NASH (mean difference = − 3.8* and − 7 * g, respectively, p-values < 0.01). Comparing 3-week and 12-week Treated with 9-week NASH group animals showed a significant decrease in liver weight (Mean difference = − 5.4* and − 5.2 * g, respectively, p-values < 0.01). However, liver weight in rats treated with the probiotic was still significantly higher than in controls (p < 0.05) (Fig. 4B). Final body weight was also positively correlated with liver weight in 12- and 3-week treated rats (r = 0.9*, p < 0.01).

### Probiotic prevented biochemical and histopathological changes of NAFLD

Probiotic decreased NASH changes in both blood and liver samples. Hepatocellular damage indicators regressed in Treated group rats; a significant decrease in AST, ALT, GGT, ALP, TB, and DB serum levels was observed comparing 12-week Treated animals to 12-week NASH group rats (mean difference = − 84.8, − 101, − 49.5, − 0.56 and − 0.21, *p*l < 0.001, except 0.19 and 0.006 for TB and DB, respectively). Probiotic normalized lipid profiles. Both 3- and 12-week treated animals showed a significant decrease in serum levels of TGs, total cholesterol, and LDL. Also, a significant increase in HDL levels was found relative to 12-week NASH group rats (*p* < 0.001) (Table 1). Liver examination in Treated group rats showed a shiny polished appearance similar to controls liver. Probiotic decreased the amount of steatosis, inflammation and fibrosis in treated rats. Although histopathological effect of NASH was minimized, residual fibrosis and ballooning of hepatocytes were still present (Table 1and Fig. 5). Immunohistochemical analysis showed that probiotic administration minimized the effect of NASH on HSC. More inactive HSCs were present in treated groups as indicated by significant decrease in α-SMA expression in both 12-week and 3-week Treated group animals. Concurrently, they showed an increase in the LATS2 expression compared to the NASH-12-week group rats (Fig. 6).

### Probiotic administration eliminated the effects of NAFLD on expression of FOXA2, TEAD2, LATS2 mRNAs, miR650, and RPARP AS-1 LncRNA

Probiotic administration eliminated the effects of NASH on expression of FOXA2, TEAD2, LATS2 mRNAs, miR650, and RPARP AS-1 LncRNA; In 3- and 12-week treated group animals showed a significant decrease in RQ expression of FOXA2, TEAD2 mRNAs and miR-650 (p < 0.01), with a significant increase in RQ expression of LATS2 mRNA and RPARP AS-1 LncRNA (p < 0.01) when compared to 12-week NASH groups rats (Fig. 7).

### Probiotic administration eliminated the effects of NAFLD on concentrations of target effector proteins, IL6 and TGF-β

Probiotic administration decreased IL6 and TGF-β concentrations. 12-week Treated group rats. IL6 and TGF-β concentrations significantly decreased, 2- and 2.2-fold (p < 0.01) compared to 12-week NASH rats. Concentrations decreased in 3-week Treated group animals similarly, 1.6- and 2-fold (p < 0.01), compared to 12-week NASH rats (Fig. 8).

## Discussion

The obesity pandemic and the concurrent increase in NAFLD/NASH prevalence continues, with NAFLD/NASH now considered the top reason for liver transplantation [43]. Still, no treatment approved by the FDA is available [44]. Imbalance in gut microbiota is proposed as a basis of NAFLD/NASH pathogenesis [27]. This explanation highlights the need to exploit probiotics to reverse gut dysbiosis both for prevention of and therapy for NAFLD/NASH [28].

Induction of NASH in rats in this study was successful, as evidenced by abnormal liver function tests, lipid profiles, and hepatic histopathological features characteristics of NAFLD/NASH macro- and micro-vesicular steatosis, with variable degrees of inflammation, and increased fibrosis. Mutaflor^®^ administration induced improvements in liver function tests and lipid profiles. Treated rats showed a marked decrease in steatosis, inflammation, and fibrosis.

The pathogenesis of NAFLD/NASH involves a constellation of insults [45] that initiates a chronic inflammatory process with hepatocytic injury, activation of immune cells [46], and stimulation of regenerative processes [45]. Regenerative is primarily mediated by HSCs [46]. Activation of HSCs induces fibrillary collagens and α-SMA, leading to extracellular matrix deposition [43]. Hh signaling is a major regulator of liver regeneration and controls the fate of HSCs [15]. Sustained and excessive activation of the Hh pathway in NAFLD is pivotal for driving the progression to fibrosing NASH [47], liver cirrhosis, and hepatocellular carcinoma [48].

We used in silico analysis to construct a genetic (FOXA2, TEAD2, LATS2) and epigenetic network (miR-650 and RPARP AS-1 LncRNA) linked to the stimulation of hedgehog and hippo signaling with subsequent activation of HSCs and its effector proteins (L-6, and TGF-β) in NAFLD/NASH. We then validated the expression of this network in liver tissue samples of a NASH animal model. We observed altered expression in the constructed network and its effector proteins in NASH hepatic tissues, accompanied by a highly significant increase in the expression of (FOXA2, TEAD2 mRNAs, miR-650), proteins (IL-6 and TGF-β) and downregulation of LATS2 mRNA and RPARP AS-1 LncRNA. We also validated the effect of Mutaflor^®^ on the network. Administration of this probiotic modulated expression of the network towards a normal expression pattern in NASH 12-week model rats.

*FOXA2*, also known as hepatocyte nuclear factor 3-Β and transcription factor 3B [49], is located on chromosome 20:22, 580, 998-22, 585, 455 reverse strand [50]. The *FOXA* family of evolutionarily conserved liver enriched transcription factors [50, 51] participate in hepatic development and differentiation [52]. *FOXA2* is essential for Hh-induced hepatic progenitor cell specification during embryogenesis [51]. We measured FOXA2 mRNA as a marker for the Hh pathway. Similarly, a study by Wang et al. showed that the FOXA2 is also a target of Hh signaling, but in the esophageal epithelium of mouse embryos [53]. The protective effect of FOXA2 against CCL4-induced hepatic fibrosis in mice, was attributed to FOXA2 downregulation in hepatocytes. However, the same study reported no correlation between upregulated FOXA2 in activated HSCs and hepatic fibrosis [54]. Nevertheless, the importance of HSCs in the development of fibrosis seems established [55–57].

*TEAD2* is a protein-coding gene located on chromosome 19:49, 340, 595-49, 362, 457 [58]. The *TEAD2* gene is considered an oncogene, where stability of its mRNA is affected in multiple tumors [56, 59, 60]. Joo et al. showed increased TEAD2 mRNA expression in HCC has a poor prognosis [61]. In NAFLD, TEAD2 is a downstream effector molecule in the hippo pathway strongly associated with HSC activation [62]. *LATS2* is a hippo signaling pathway kinase that plays an important inhibitory role during the differentiation and maturation of the liver [19]. Deletion of LATS2 in adult liver is associated with fibrosis and even lethal hepatic impairment [63]. Conversely, *LATS2* inhibits hepatic cholesterol accumulation in the liver [64], explaining the increase in fatty liver incidence in LATS2-deficient mice [65]. Ye et al. showed that direct inhibition of LATS2 activity participates in increasing the progression of NASH to HCC [66].

IL-6, a pleiotropic 184 amino acid cytokine that has a significant role in the regeneration of the liver [21] and is a potent stimulant of hepatocyte proliferation. Additionally, IL-6 increases the expression of fibrogenesis genes in the liver [67]. HSCs are directly affected by IL-6 signaling, since they are one of the few cells that express IL-6 membrane-bound receptors [68]. Also, HSCs secrete IL-6, which eventually lead to an increase in type I collagen production and thus fibrosis in culture [62]. Accumulating evidence identifies a leading role of the IL-6 pathway in HSC activation, which is critical for the progression of liver fibrosis [69–73]. TGF-β is the most potent fibrogenic cytokine in the liver [20]. An increase in TGF-β expression in active HSCs was reported after liver injury [74]. Moreover, Dewair et al*.* identified TGF-β signaling as a key fibrogenic pathway that drives activation of HSC and fibrosis in liver disease [10]. Experimentally, hepatic fibrosis was decreased on inhibiting TGF-β activity in rats [75]. The results of our study are consistent with findings in all of the above studies.

Epigenetics play a key role in the pathogenesis of NAFLD, indicating its importance as a possible therapeutic target [22]. Our in silico model identified miR-650 and RPARP-AS1 LncRNA as significant epigenetic regulators of selected mRNAs. miR-650 is implicated in fibrosis due to its upregulation in idiopathic lung fibrosis [76]. Estep et al. found significant downregulation of hsa-miR-650 expression in visceral adipose tissue of NASH patients compared with patients with non-NASH NAFLD, demonstrating its role in liver fibrosis [77]. miR-650 is implicated in inflammation and apoptosis in active ulcerative colitis [78]. Han et al. identified LATS2 as a direct target of miR-650; LATS2 can counteract the effects of miR-650 in HCC [79]. Similarly, Zhou et al. demonstrated a key role for LncRNAs in HSCs activation, thus increasing fibrosis in liver disease [80]. We identified RPARP-AS1 LncRNA as a novel factor that interacts with miR-650. The antisense LncRNA is located on the 10q24.32 chromosome [81] implicated in promotion of cell proliferation, migration, and invasion in colorectal cancer [82].

Activated HSCs trans-differentiate into contractile fibrogenic myofibroblast cells (MF) that express α-SMA [83]. An interaction between HSCs and virally-infected hepatocytes increases the deleterious anti-viral inflammatory response in hepatitis C infection [84]. However, active HSCs after acute liver injury exhibit a protective role by assisting in extracellular matrix deposition and inflammatory signaling [85]. Thus, we considered active HSCs as our main therapeutic target, recognizing its role in the pathogenesis of NAFLD/NASH. Consistently, our results show a marked increase in α-SMA expression as an indicator of active HSCs in NASH model rats, which is reversed to normal by administration of Mutaflor® and accompanied by a decrease in the number of active HSCs.

**Fig. 9:**
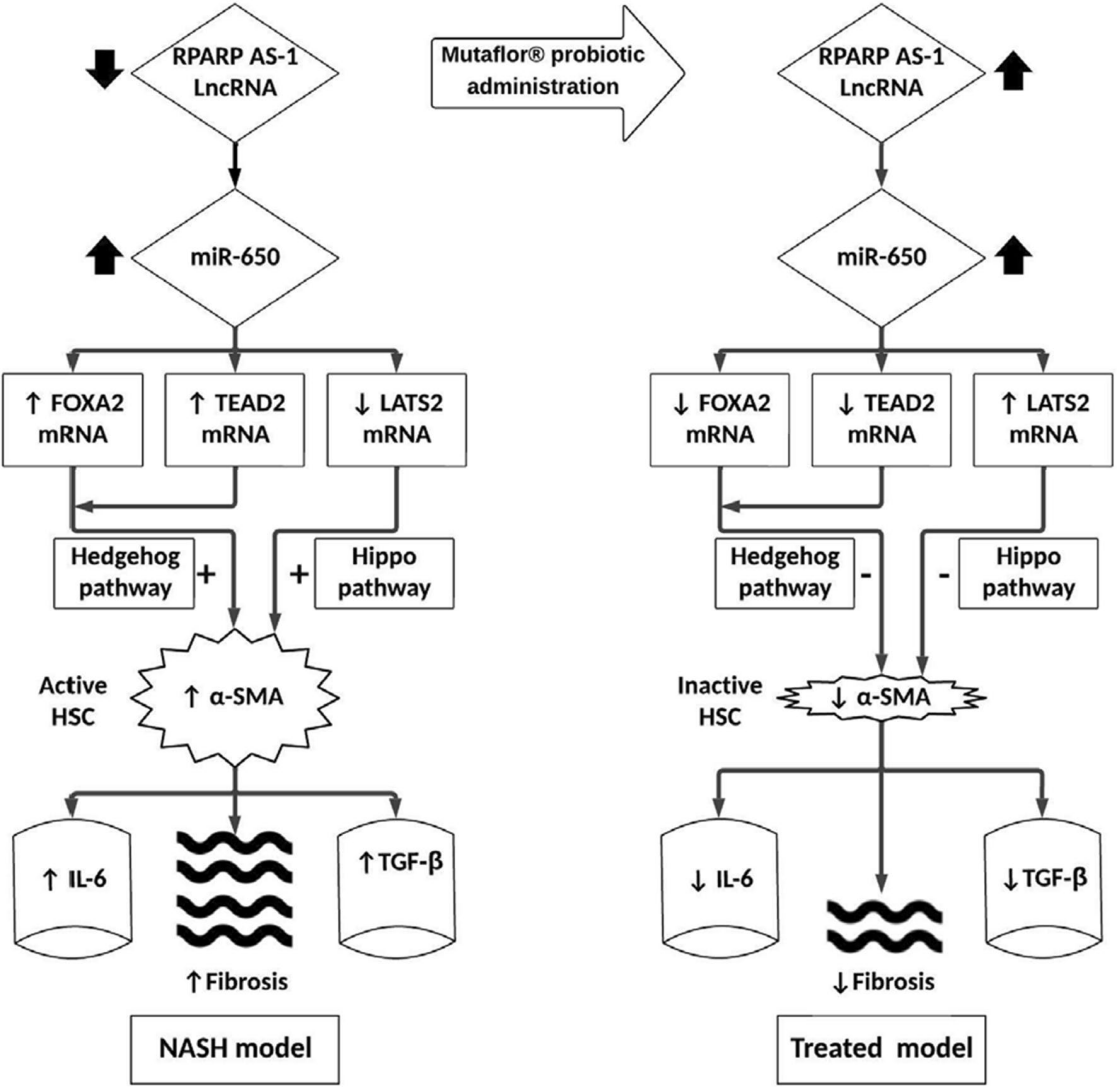
Proof of Concept map of the study hypothesis

A significant strong positive correlation was seen among FOXA2, TEAD2 mRNAs, miR-650, IL-6 and TGF-β effector proteins, along with a significant negative correlation with LATS2 mRNA and RPARP AS1-LncRNA. We hypothesized that, in NAFLD/NASH, increased expression of miR-650 upregulates both FOXA2 and TEAD2 mRNAs and downregulates LATS2 mRNA. Stimulating both hedgehog and hippo signaling, activating HSCs and increasing HSC expression of profibrogenic signals (IL-6 and TGF-β effector proteins). Consequently increasing hepatic fibrosis and NAFLD/NASH progression. On Mutaflor^®^ administration, increased expression RPARP AS-1 LncRNA inhibits miR-650. This downregulates FOXA2 and TEAD2 mRNAs and upregulates LATS mRNA. Inhibiting both hedgehog and hippo signaling, inactivating HSCs and decreasing HSC expression of profibrogenic signals (IL-6 and TGF-β effector proteins). Eventually, the probiotic decrease hepatic fibrosis and NAFLD/NASH progression (Fig. 9).

## Conclusion

The use of probiotics clinical trials are promising. In NAFLD, stopping HSCs activation will halt progression and regress existing fibrosis. As stated, HSCs activity is controlled by Gut dysbiosis. As a result, using probiotic to target HSCs may be a potential preventive and treatment strategy in NAFLD. In this study we concluded that Mutaflor® shows a significant ability to counter the activation of HSCs and decrease HSC upregulation of fibrinogenic genes (IL-6, and TGF-β proteins). These actions are associated with modulating hedgehog and hippo signaling pathways via FOXA2, TEAD2 and LATS2 mRNAs, and their epigenetic modifiers, (RPARP AS-1 and miR-650 miRNA). This probiotic agent show significant improvement in NAFLD animal model suggesting its usefulness for prevention and treatment of NAFLD/NASH.

## Limitations

Limitation of the present study includes the lack of data regarding the rats’ physical activity and energy expenditure, which could compensate for HSHFD consumed by the rats. Future studies should measure rat activity, which can then be correlated to any histopathological changes in the liver. Second, studying the effect of silencing of one of the explored genetic network on the pathogenesis of NASH. Finally, although probiotic was studied as a NASH management tool in rats, it is essential to highlight that translating animal-study outcomes to the human population has a failure history [86]. So more randomized clinical studies with extended follow-up can illuminate the effects of probiotics in prevention and therapy of NASH in human.

## Supplementary Information

**Additional file 1**: **figures S1–S7**. Database screen shots for retrieved and validated data demonstrating each step of the bioinformatics workflow.

## Data Availability

All data are available upon request.

## Abbreviations

ALP: Alkaline phosphatase
AST: Aspartate transaminase
ALT: Alanine transaminase
α-SMA: Alpha-smooth muscle actin
CFU/mL: Colony-forming units per mL
DAB: Diaminobenzidine
DB: Direct bilirubin
ELISA: Enzyme-linked immunosorbent assay
EcN: *Escherichia coli* Nissle
Foxa2: Forkhead box A2
FMASU: Faculty Of Medicine Ain Shams University
GGT: Gamma-glutamyl transferase
HDL: High-density lipoprotein
Hh: Hedgehog
H&E: Hematoxylin and eosin
HSCs: Hepatic stellate cells
HCC: Hepatocellular carcinoma
HFHSD: High-fat high sucrose diet
H2O2: Hydrogen peroxide
LATS2: Large tumor suppressor kinase 2
LB: Luria Bertani
IL-6: Interleukin-6
LDL: Low-density lipoprotein
LncRNA: Long noncoding RNA
miRs: MicroRNAs
miR-650: MicroRNA 650
mL: Milliliter
mRNAs: Messenger RNAs
NAFLD: Nonalcoholic fatty liver disease
NASH: Nonalcoholic steatohepatitis
PBS: Phosphate buffered saline
REC: Research Ethics Committee
Rpm: Rate per minute
RT-qPCR: Real-time quantitative Polymerase chain reaction
TB: Total bilirubin
Tc: Total cholesterol
TEAD2: TEA domain transcription factor 2
TGF-β: Transforming growth factor β
TGs: Triglycerides

## References

[1] Pydyn N, Miękus K, Jura J, Kotlinowski J (2020). New therapeutic strategies in nonalcoholic fatty liver disease: a focus on promising drugs for nonalcoholic steatohepatitis. Pharmacol Rep.

[2] Perumpail BJ, Khan MA, Yoo ER, Cholankeril G, Kim D, Ahmed A (2017). Clinical epidemiology and disease burden of nonalcoholic fatty liver disease. World J Gastroenterol.

[3] Negro F (2020). Natural history of NASH and HCC. Liver Int.

[4] Burra P, Becchetti C, Germani G (2020). NAFLD and liver transplantation: disease burden, current management and future challenges. JHEP Rep.

[5] Fang YL, Chen H, Wang CL, Liang L (2018). Pathogenesis of non-alcoholic fatty liver disease in children and adolescence: from "two hit theory" to "multiple hit model". World J Gastroenterol.

[6] Li X, Zhu L, Wang B, Yuan M, Zhu R (2017). Drugs and targets in fibrosis. Front Pharmacol.

[7] Zisser A, Ipsen DH, Tveden-Nyborg P (2021). Hepatic stellate cell activation and inactivation in NASH-fibrosis-roles as putative treatment targets?. Biomedicines.

[8] Schwabe RF, Tabas I, Pajvani UB (2020). Mechanisms of fibrosis development in nonalcoholic steatohepatitis. Gastroenterology.

[9] Friedman SL, Neuschwander-Tetri BA, Rinella M, Sanyal AJ (2018). Mechanisms of NAFLD development and therapeutic strategies. Nat Med.

[10] Dewidar B, Meyer C, Dooley S, Meindl-Beinker AN (2019). TGF-β in hepatic stellate cell activation and liver fibrogenesis—updated 2019. Cells.

[11] Marcher AB, Bendixen SM, Terkelsen MK, Hohmann SS, Hansen MH, Larsen BD, Mandrup S, Dimke H, Detlefsen S, Ravnskjaer K (2019). Transcriptional regulation of hepatic stellate cell activation in NASH. Sci Rep.

[12] Heyens LJM, Busschots D, Koek GH, Robaeys G, Francque S (2021). Liver fibrosis in non-alcoholic fatty liver disease: from liver biopsy to non-invasive biomarkers in diagnosis and treatment. Front Med.

[13] Kiagiadaki F, Kampa M, Voumvouraki A, Castanas E, Kouroumalis E, Notas G (2018). Activin-A causes hepatic stellate cell activation via the induction of TNFα and TGFβ in Kupffer cells. Biochim Biophys Acta Mol Basis Dis.

[14] Chan YT, Wang N, Tan HY, Li S, Feng Y (2020). Targeting hepatic stellate cells for the treatment of liver fibrosis by natural products: is it the dawning of a new era?. Front Pharmacol.

[15] Hou W, Syn WK (2018). Role of metabolism in hepatic stellate cell activation and fibrogenesis. Front Cell Dev Biol.

[16] Gao L, Zhang Z, Zhang P, Yu M, Yang T (2018). Role of canonical Hedgehog signaling pathway in liver. Int J Biol Sci.

[17] Mu T, Xu L, Zhong Y, Liu X, Zhao Z, Huang C, Lan X, Lufei C, Zhou Y, Su Y, Xu L, Jiang M, Zhou H, Lin X, Wu L, Peng S, Liu S, Brix S, Dean M, Dunn NR, Zaret KS, Fu XY, Hou Y (2020). Embryonic liver developmental trajectory revealed by single-cell RNA sequencing in the Foxa2eGFP mouse. Commun Biol.

[18] Manmadhan S, Ehmer U (2019). Hippo signaling in the liver—a long and ever-expanding story. Front Cell Dev Biol.

[19] Yi J, Lu L, Yanger K, Wang W, Sohn BH, Stanger BZ, Zhang M, Martin JF, Ajani JA, Chen J, Lee JS, Song S, Johnson RL (2016). Large tumor suppressor homologs 1 and 2 regulate mouse liver progenitor cell proliferation and maturation through antagonism of the coactivators YAP and TAZ. Hepatology.

[20] Tsuchida T, Friedman SL (2017). Mechanisms of hepatic stellate cell activation. Nat Rev Gastroenterol Hepatol.

[21] Naseem S, Hussain T, Manzoor S (2018). Interleukin-6: a promising cytokine to support liver regeneration and adaptive immunity in liver pathologies. Cytokine Growth Factor Rev.

[22] Ashraf NU, Altaf M (2018). Epigenetics: an emerging field in the pathogenesis of nonalcoholic fatty liver disease. Mutat Res.

[23] Eslam M, Valenti L, Romeo S (2018). Genetics and epigenetics of NAFLD and NASH: clinical impact. J Hepatol.

[24] Finotti A, Fabbri E, Lampronti I, Gasparello J, Borgatti M, Gambari R (2019). MicroRNAs and long non-coding RNAs in genetic diseases. Mol Diagn Ther.

[25] Lin HY, Yang YL, Wang PW, Wang FS, Huang YH (2020). The emerging role of microRNAs in NAFLD: highlight of microRNA-29a in modulating oxidative stress, inflammation, and beyond. Cells.

[26] Hanson A, Wilhelmsen D, DiStefano JK (2018). The role of long non-coding RNAs (lncRNAs) in the development and progression of fibrosis associated with nonalcoholic fatty liver disease (NAFLD). Noncoding RNA.

[27] Iacono A, Raso GM, Canani RB, Calignano A, Meli R (2011). Probiotics as an emerging therapeutic strategy to treat NAFLD: focus on molecular and biochemical mechanisms. J Nutr Biochem.

[28] Perumpail BJ, Li AA, John N, Sallam S, Shah ND, Kwong W, Cholankeril G, Kim D, Ahmed A (2019). The therapeutic implications of the gut microbiome and probiotics in patients with NAFLD. Diseases.

[29] Meroni M, Longo M, Dongiovanni P (2019). The role of probiotics in nonalcoholic fatty liver disease: a new insight into therapeutic strategies. Nutrients.

[30] Duseja A, Acharya SK, Mehta M, Chhabra S, Rana S, Das A, Dattagupta S, Dhiman RK, Chawla YK (2019). High potency multistrain probiotic improves liver histology in non-alcoholic fatty liver disease (NAFLD): a randomised, double-blind, proof of concept study. BMJ Open Gastroenterol.

[31] Scaldaferri F, Gerardi V, Mangiola F, Lopetuso LR, Pizzoferrato M, Petito V, Papa A, Stojanovic J, Poscia A, Cammarota G, Gasbarrini A (2016). Role and mechanisms of action of *Escherichia coli* Nissle 1917 in the maintenance of remission in ulcerative colitis patients: an update. World J Gastroenterol.

[32] Wassenaar TM (2016). Insights from 100 years of research with probiotic *E. coli*. Eur J Microbiol Immunol.

[33] Hungin AP, Mulligan C, Pot B, Whorwell P, Agréus L, Fracasso P, Lionis C, Mendive J, de Foy JMP, Rubin G, Winchester C, de Wit N (2013). Systematic review: probiotics in the management of lower gastrointestinal symptoms in clinical practice—an evidence-based international guide. Aliment Pharmacol Ther.

[34] Praveschotinunt P, Duraj-Thatte AM, Gelfat I, Bahl F, Chou DB, Joshi NS (2019). Engineered *E. coli* Nissle 1917 for the delivery of matrix-tethered therapeutic domains to the gut. Nat Commun.

[35] Reynolds J, Farinha M. Counting bacteria. Biology 2420 laboratory manual: microbiology. Richland College, Dallas, USA. 2005;1–10.

[36] Kosakova D, Scheer P, Lata J, Doubek J (2007). Influence of the *Escherichia coli* Nissle 1917 strain on complications of the chronic experimental liver damage. Vet Med.

[37] Arribas B, Rodríguez-Cabezas ME, Camuesco D, Comalada M, Bailón E, Utrilla P, Nieto A, Concha A, Zarzuelo A, Gálvez J (2009). A probiotic strain of *Escherichia coli*, Nissle 1917, given orally exerts local and systemic anti-inflammatory effects in lipopolysaccharide-induced sepsis in mice. Br J Pharmacol.

[38] Briskey D, Heritage M, Jaskowski LA, Peake J, Gobe G, Subramaniam VN, Crawford D, Campbell C, Vitetta L (2016). Probiotics modify tight-junction proteins in an animal model of nonalcoholic fatty liver disease. Therap Adv Gastroenterol.

[39] Sha S, Xu B, Kong X, Wei N, Liu J, Wu K (2014). Preventive effects of *Escherichia coli* strain Nissle 1917 with different courses and different doses on intestinal inflammation in murine model of colitis. Inflamm Res.

[40] Guo JH, Han DW, Li XQ, Zhang Y, Zhao YC (2014). The impact of small doses of LPS on NASH in high sucrose and high fat diet induced rats. Eur Rev Med Pharmacol Sci.

[41] Khatun S, Fujimoto J, Toyoki H, Tamaya T (2003). Clinical implications of expression of ETS-1 in relation to angiogenesis in ovarian cancers. Cancer Sci.

[42] Takahashi Y, Fukusato T (2014). Histopathology of nonalcoholic fatty liver disease/nonalcoholic steatohepatitis. World J Gastroenterol.

[43] Paul J (2020). Recent advances in non-invasive diagnosis and medical management of non-alcoholic fatty liver disease in adult. Egypt Liver J.

[44] Wong VW, Singal AK (2019). Emerging medical therapies for non-alcoholic fatty liver disease and for alcoholic hepatitis. Transl Gastroenterol Hepatol.

[45] Kaufmann B, Reca A, Wang B, Friess H, Feldstein AE, Hartmann D (2021). Mechanisms of nonalcoholic fatty liver disease and implications for surgery. Langenbecks Arch Surg.

[46] Higashi T, Friedman SL, Hoshida Y (2017). Hepatic stellate cells as key target in liver fibrosis. Adv Drug Deliv Rev.

[47] Della Corte CM, Viscardi G, Papaccio F, Esposito G, Martini G, Ciardiello D, Martinelli E, Ciardiello F, Morgillo F (2017). Implication of the Hedgehog pathway in hepatocellular carcinoma. World J Gastroenterol.

[48] Mouse (GRCm39) Ensembl release 103—February 2021 © EMBL-EBI: ENSMUSG00000037025. http://www.ensembl.org/Mus_musculus/Info/Index?db=core;g=ENSMUSG00000037025;r=2:147884797-147888889. Accessed July 2021.

[49] Human (GRCh38) Ensembl release 103 - February 2021 © EMBL-EBI: ENSG00000125798. http://www.ensembl.org/Homo_sapiens/Info/Index?db=core;g=ENSG00000125798;r=20:22580998-22585455. Accessed July 2021.

[50] Golson ML, Kaestner KH (2016). Fox transcription factors: from development to disease. Development.

[51] Bernardo GM, Keri RA (2012). FOXA1: a transcription factor with parallel functions in development and cancer. Biosci Rep.

[52] Omenetti A, Choi S, Michelotti G, Diehl AM (2011). Hedgehog signaling in the liver. J Hepatol.

[53] Wang DH, Tiwari A, Kim ME, Clemons NJ, Regmi NL, Hodges WA, Berman DM, Montgomery EA, Watkins DN, Zhang X, Zhang Q, Jie C, Spechler SJ, Souza RF (2014). Hedgehog signaling regulates FOXA2 in esophageal embryogenesis and Barrett’s metaplasia. J Clin Investig.

[54] Wang W, Yao LJ, Shen W, Ding K, Shi PM, Chen F, He J, Ding J, Zhang X, Xie WF (2017). FOXA2 alleviates CCl4-induced liver fibrosis by protecting hepatocytes in mice. Sci Rep.

[55] Wilhelm A, Aldridge V, Haldar D, Naylor AJ, Weston CJ, Hedegaard D, Garg A, Fear J, Reynolds GM, Croft AP, Henderson NC, Buckley CD, Newsome PN (2016). CD248/endosialin critically regulates hepatic stellate cell proliferation during chronic liver injury via a PDGF-regulated mechanism. Gut.

[56] Henderson NC, Arnold TD, Katamura Y, Giacomini MM, Rodriguez JD, McCarty JH, Pellicoro A, Raschperger E, Betsholtz C, Ruminski PG, Griggs DW, Prinsen MJ, Maher JJ, Iredale JP, Lacy-Hulbert A, Adams RH, Sheppard D (2013). Targeting of αv integrin identifies a core molecular pathway that regulates fibrosis in several organs. Nat Med..

[57] Chen L, Li J, Zhang J, Dai C, Liu X, Wang J, Gao Z, Guo H, Wang R, Lu S, Wang F, Zhang H, Chen H, Fan X, Wang S, Qin Z (2015). S100A4 promotes liver fibrosis via activation of hepatic stellate cells. J Hepatol.

[58] Human (GRCh38/hg38) Ensembl release 103—February 2021 © EMBL-EBI: ENSG00000074219. http://www.ensembl.org/Homo_sapiens/Gene/Summary?g=ENSG00000074219;r=19:49340595-49362457. Accessed July 2021.

[59] Zhao Z, Meng J, Su R, Zhang J, Chen J, Ma X, Xia Q (2020). Epitranscriptomics in liver disease: basic concepts and therapeutic potential. J Hepatol.

[60] Rong ZX, Li Z, He JJ, Liu LY, Ren XX, Gao J, Mu Y, Guan YD, Duan YM, Zhang XP, Zhang DX, Li N, Deng YZ, Sun LQ (2019). Downregulation of fat mass and obesity associated (FTO) promotes the progression of intrahepatic cholangiocarcinoma. Front Oncol.

[61] Joo JS, Cho SY, Rou WS, Kim JS, Kang SH, Lee ES, Moon HS, Kim SH, Sung JK, Kwon IS, Eun HS, Lee BS (2020). TEAD2 as a novel prognostic factor for hepatocellular carcinoma. Oncol Rep.

[62] Shu Y, Liu X, Huang H, Wen Q, Shu J (2021). Research progress of natural compounds in anti-liver fibrosis by affecting autophagy of hepatic stellate cells. Mol Biol Rep.

[63] Lee DH, Park JO, Kim TS, Kim SK, Kim TH, Kim MC, Park GS, Kim JH, Kuninaka S, Olson EN, Saya H, Kim SY, Lee H, Lim DS (2016). LATS-YAP/TAZ controls lineage specification by regulating TGFβ signaling and Hnf4α expression during liver development. Nat Commun.

[64] Aylon Y, Gershoni A, Rotkopf R, Biton IE, Porat Z, Koh AP, Sun X, Lee Y, Fiel MI, Hoshida Y, Friedman SL, Johnson RL, Oren M (2016). The LATS2 tumor suppressor inhibits SREBP and suppresses hepatic cholesterol accumulation. Genes Dev.

[65] Furth N, Aylon Y (2017). The LATS1 and LATS2 tumor suppressors: beyond the Hippo pathway. Cell Death Differ.

[66] Ye J, Li TS, Xu G, Zhao YM, Zhang NP, Fan J, Wu J (2017). JCAD promotes progression of nonalcoholic steatohepatitis to liver cancer by inhibiting LATS2 kinase activity. Cancer Res.

[67] Schmidt-Arras D, Rose-John S (2016). IL-6 pathway in the liver: From physiopathology to therapy. J Hepatol.

[68] Fazel Modares N, Polz R, Haghighi F, Lamertz L, Behnke K, Zhuang Y, Kordes C, Häussinger D, Sorg UR, Pfeffer K, Floss DM, Moll JM, Piekorz RP, Ahmadian MR, Lang PA, Scheller J (2019). IL-6 trans-signaling controls liver regeneration after partial hepatectomy. Hepatology.

[69] Xiang DM, Sun W, Ning BF, Zhou TF, Li XF, Zhong W, Cheng Z, Xia MY, Wang X, Deng X, Wang W, Li HY, Cui XL, Li SC, Wu B, Xie WF, Wang HY, Ding J (2018). The HLF/IL-6/STAT3 feedforward circuit drives hepatic stellate cell activation to promote liver fibrosis. Gut.

[70] Wang H, Lafdil F, Kong X, Gao B (2011). Signal transducer and activator of transcription 3 in liver diseases: a novel therapeutic target. Int J Biol Sci.

[71] Kong X, Horiguchi N, Mori M, Gao B (2012). Cytokines and STATs in liver fibrosis. Front Physiol.

[72] Granzow M, Schierwagen R, Klein S, Kowallick B, Huss S, Linhart M, Mazar IG, Görtzen J, Vogt A, Schildberg FA, Gonzalez-Carmona MA, Wojtalla A, Krämer B, Nattermann J, Siegmund SV, Werner N, Fürst DO, Laleman W, Knolle P, Shah VH, Sauerbruch T, Trebicka J (2014). Angiotensin-II type 1 receptor-mediated Janus kinase 2 activation induces liver fibrosis. Hepatology.

[73] Lakner AM, Moore CC, Gulledge AA, Schrum LW (2010). Daily genetic profiling indicates JAK/STAT signaling promotes early hepatic stellate cell transdifferentiation. World J Gastroenterol.

[74] Fabregat I, Caballero-Díaz D (2018). Transforming growth factor-β-induced cell plasticity in liver fibrosis and hepatocarcinogenesis. Front Oncol.

[75] Liu J, Kong D, Qiu J, Xie Y, Lu Z, Zhou C, Liu X, Zhang R, Wang Y (2019). Praziquantel ameliorates CCl4-induced liver fibrosis in mice by inhibiting TGF-β/Smad signalling via up-regulating Smad7 in hepatic stellate cells. Br J Pharmacol.

[76] Rajasekaran S, Rajaguru P, Sudhakar Gandhi PS (2015). MicroRNAs as potential targets for progressive pulmonary fibrosis. Front Pharmacol.

[77] Estep M, Armistead D, Hossain N, Elarainy H, Goodman Z, Baranova A, Chandhoke V, Younossi ZM (2010). Differential expression of miRNAs in the visceral adipose tissue of patients with non-alcoholic fatty liver disease. Aliment Pharmacol Ther.

[78] Fisher K, Lin J (2015). MicroRNA in inflammatory bowel disease: translational research and clinical implication. World J Gastroenterol.

[79] Han LL, Yin XR, Zhang SQ (2018). miR-650 promotes the metastasis and epithelial-mesenchymal transition of hepatocellular carcinoma by directly inhibiting LATS2 expression. Cell Physiol Biochem.

[80] Zhou C, York SR, Chen JY, Pondick JV, Motola DL, Chung RT, Mullen AC (2016). Long noncoding RNAs expressed in human hepatic stellate cells form networks with extracellular matrix proteins. Genome Med.

[81] Human (GRCh38.p13) Ensembl release 103—February 2021 © EMBL-EBI: ENSG00000269609. http://www.ensembl.org/Homo_sapiens/Gene/Summary?g=ENSG00000269609;r=10:102449816-102461106#.

[82] Ren Y, Zhao C, He Y, Min X, Xu H, Hu X (2021). RPARP-AS1/miR125a-5p axis promotes cell proliferation, migration and invasion in colon cancer. Onco Targets Ther.

[83] Khomich O, Ivanov AV, Bartosch B (2019). Metabolic hallmarks of hepatic stellate cells in liver fibrosis. Cells.

[84] Li H, Huang MH, Jiang JD, Peng ZG (2018). Hepatitis C: from inflammatory pathogenesis to anti-inflammatory/hepatoprotective therapy. World J Gastroenterol.

[85] Li J, Zhao YR, Tian Z (2019). Roles of hepatic stellate cells in acute liver failure: from the perspective of inflammation and fibrosis. World J Hepatol.

[86] Van Herck MA, Vonghia L, Francque SM (2017). Animal models of nonalcoholic fatty liver disease—a starter’s guide. Nutrients.

