# Supplementary material for: Modulation of hepatic stellate cells by Mutaflor® probiotic in non-alcoholic fatty liver disease management
Source: J Transl Med. 2022 Jul 30;20:342. doi: 10.1186/s12967-022-03543-z (PMC9338485; doi:10.1186/s12967-022-03543-z)
Supplement: Supplementary file 1 — Additional file 1: figures S1–S7. Database screen shots for retrieved and validated data demonstrating each step of the bioinformatics workflow. [file 12967_2022_3543_MOESM1_ESM.docx]

# **Modulation of hepatic stellate cells by Mutaflor® probiotic in non-alcoholic fatty liver disease management**

Supplementary figures


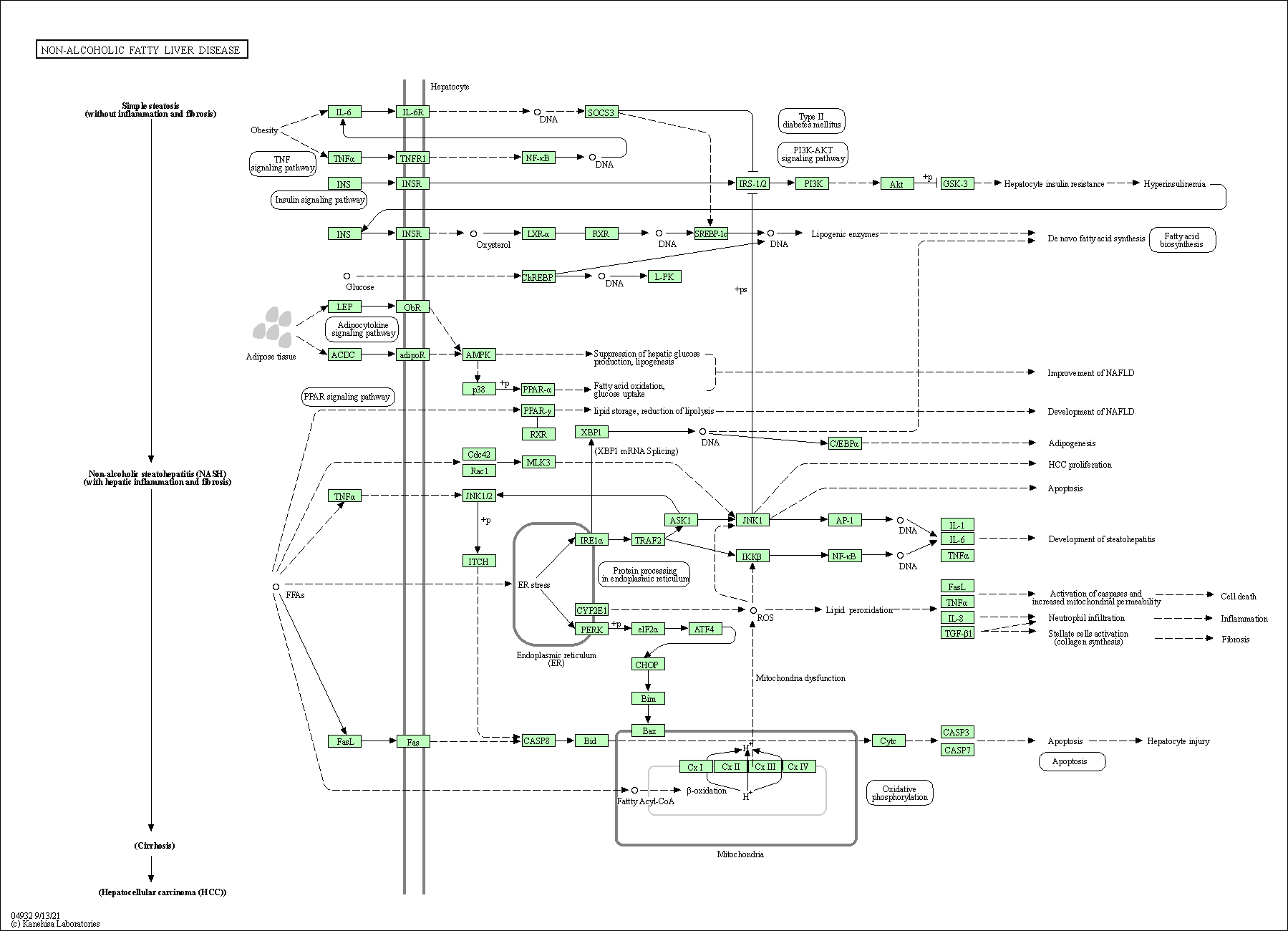


**Fig. S1A:** Print screen showing involvement of Hepatic stellate cells, TGF-β and IL-6 in NAFLD pathogenesis


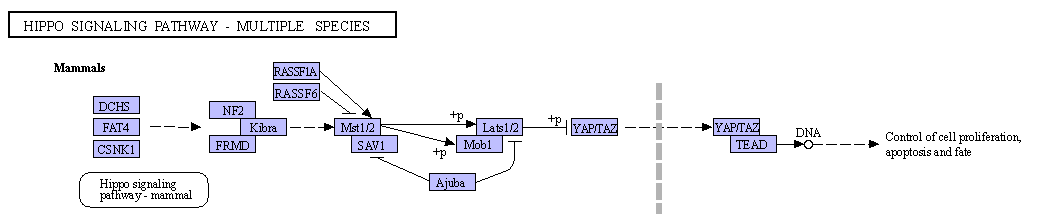
 **Fig. S1B:** Print screen showing involvement of LATS2 gene in hippo signalling available at <https://www.genome.jp/kegg-bin/show_pathway?ko04392>.


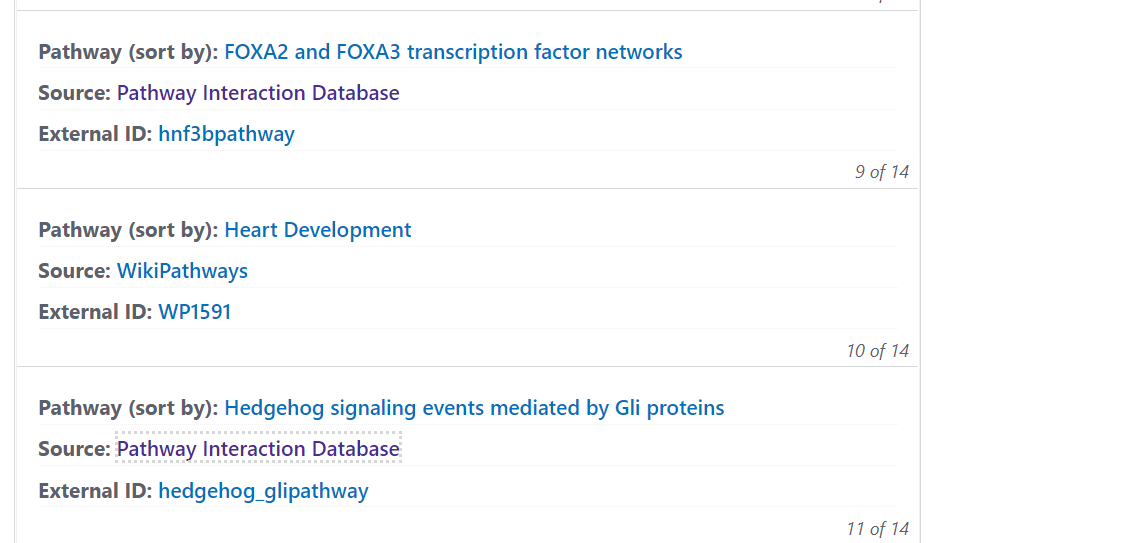
**Fig. S2A:** Print screen showing retrieval of FOXA2 from NCBI available at <https://www.ncbi.nlm.nih.gov/gene?Db=gene&Cmd=DetailsSearch&Term=3170>.


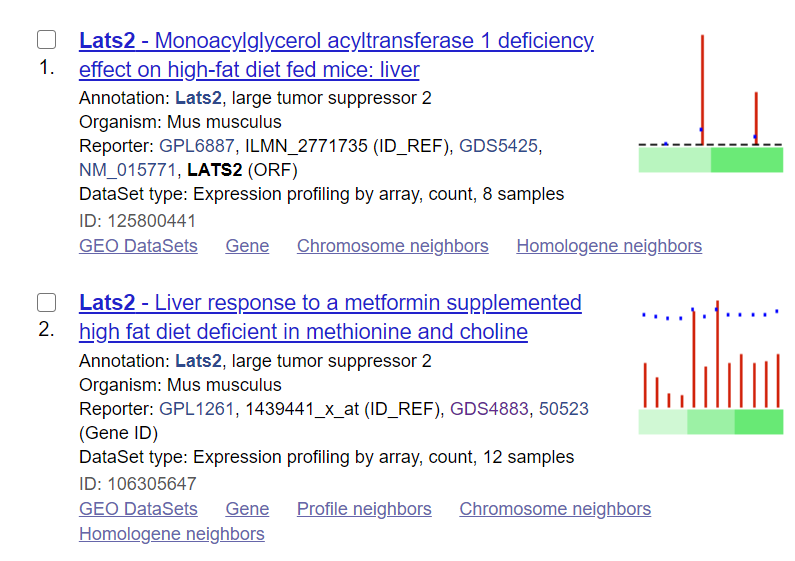
 **Fig. S2B:** Print screen showing retrieval of LATS2 from GEO available at <https://www.ncbi.nlm.nih.gov/geoprofiles/?term=LATS2+AND+NASH>.


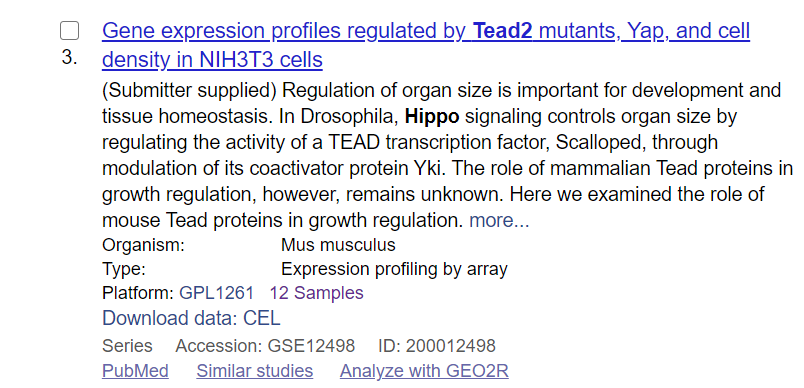
 **Fig. S2C: Print screen showing retrieval of TEAD2 from GEO available at** [**https://www.ncbi.nlm.nih.gov/geoprofiles/?term=TEAD2%2C%20NASH**](https://www.ncbi.nlm.nih.gov/geoprofiles/?term=TEAD2%2C%20NASH)**.**


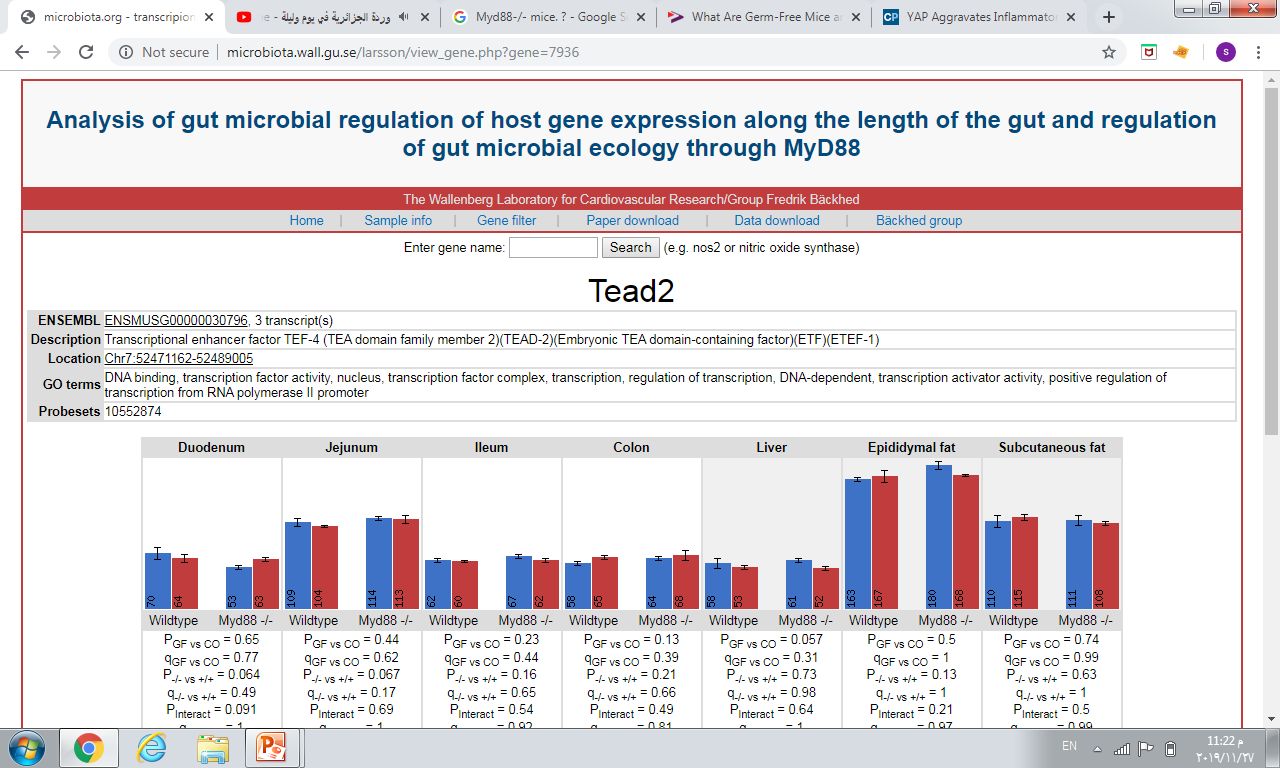


**Fig. S3A: Print screen showing that TEAD2 is one of the key regulated gut microbiota genes.** **Available at** [**http://microbiota.wall.gu.se**](http://microbiota.wall.gu.se/)


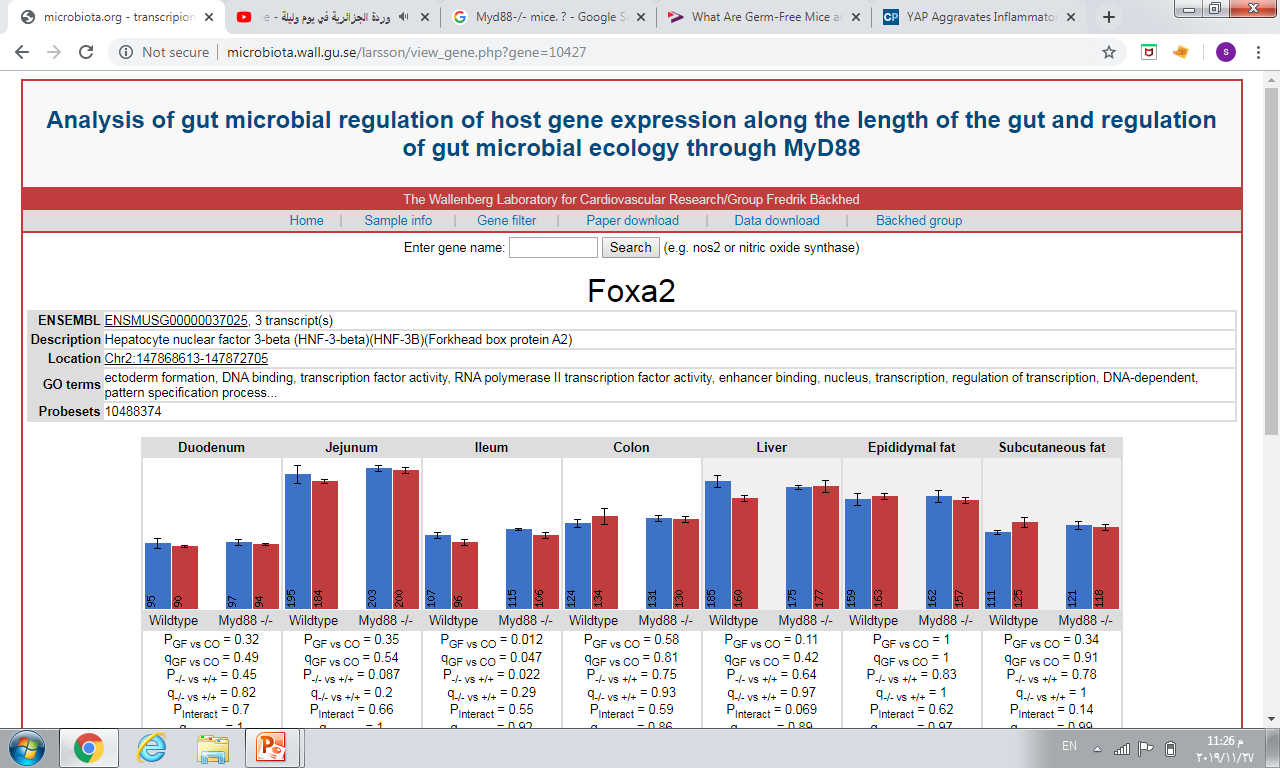
 **Fig. S3B:Print screen showing that FOXA2 one of the key regulated gut microbiota gene.** **Available at** [**http://microbiota.wall.gu.se**](http://microbiota.wall.gu.se/)**.**


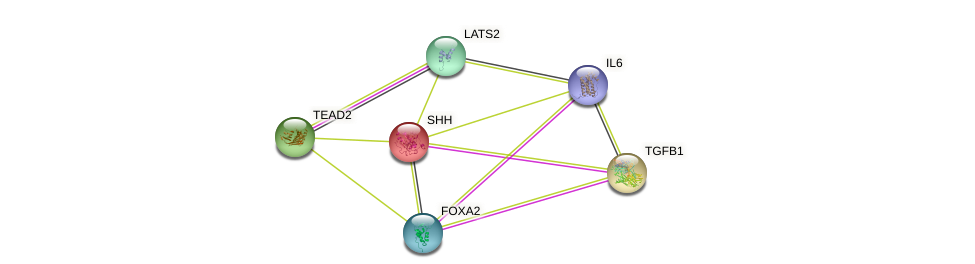
 **Fig. S4: Screen shot showing the interaction between chosen gene and target effector protein (TGFβ and IL-6) using string available at**  [**https://string-db.org/**](%20https://string-db.org/)**.**


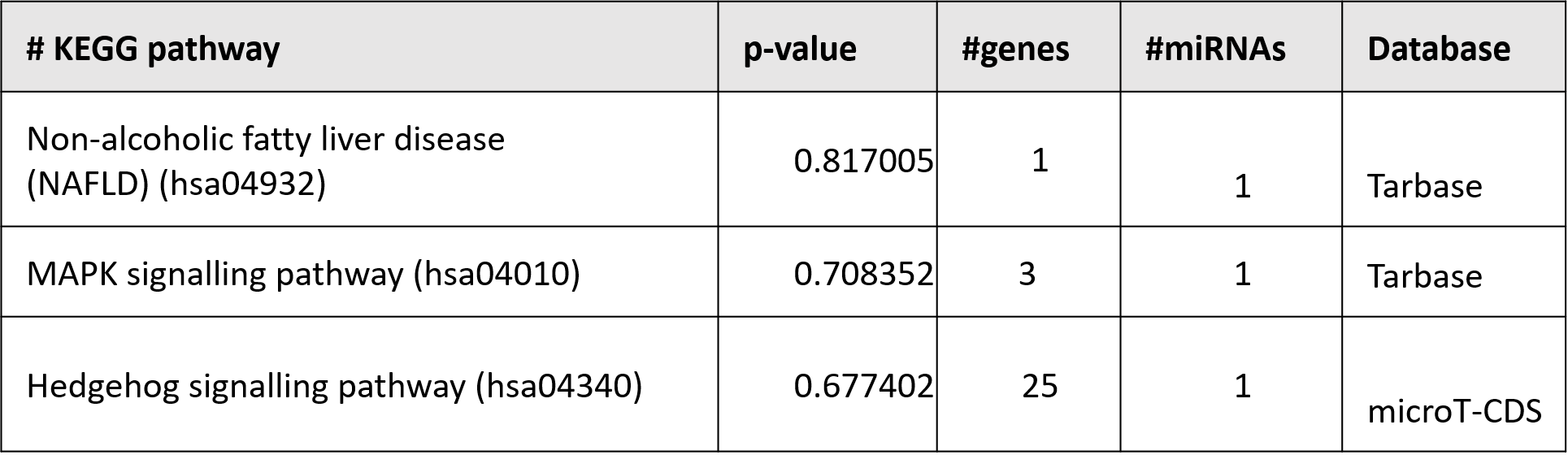


**Fig. S5: Screen shot showing enrichment of miR-650 by linking to NAFLD, MAPK and Hh signalling pathways available at** [**http://snf-515788.vm.okeanos.grnet.gr/#mirnas=hsa-miR-650;hsa-miR-650&methods=microT-CDS;Tarbase&selection=0**](http://snf-515788.vm.okeanos.grnet.gr/)**.**


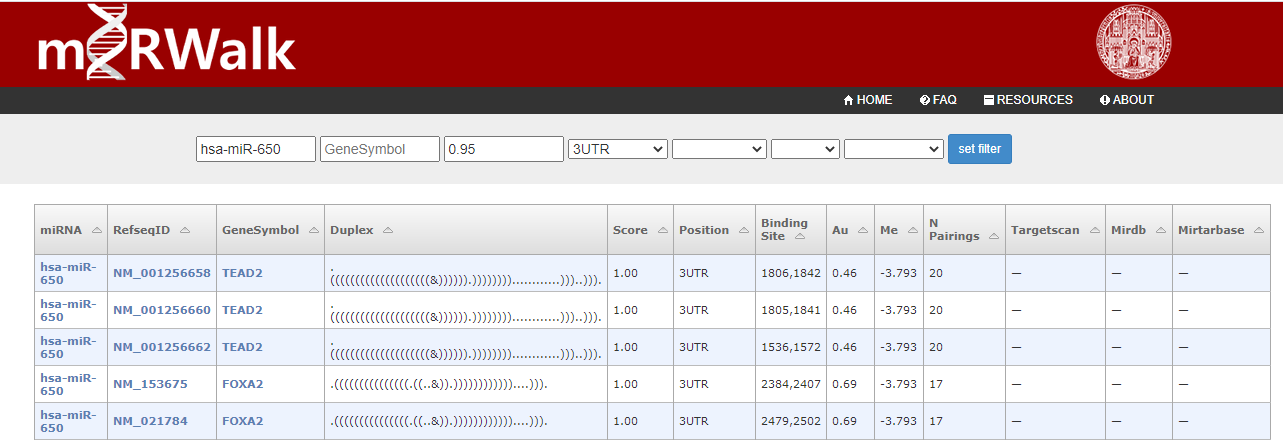
 **Fig. S6A: Print screen showing the interaction between genes and miR-650 available at** [**http://mirwalk.umm.uni-heidelberg.de/interactions/?mirnaid=hsa-miR-650&genesymbol=&bindingp=0.95&position=3UTR&targetscan=0&mirdb=0&mirtarbase=0&submit=set+filter**](http://mirwalk.umm.uni-heidelberg.de/interactions/?mirnaid=hsa-miR-650&genesymbol=&bindingp=0.95&position=3UTR&targetscan=0&mirdb=0&mirtarbase=0&submit=set+filter)**.**


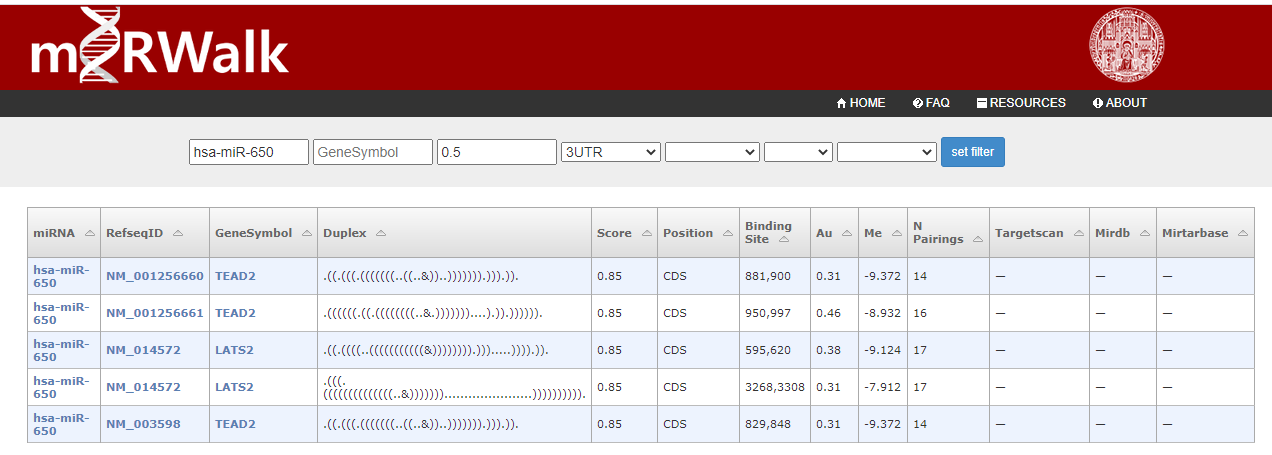
 **Fig. S6B: Print screen showing the interaction between TEAD2 AND LATS2 genes with miR-650 available at** [**http://mirwalk.umm.uni-heidelberg.de/interactions/?mirnaid=hsa-miR-650&genesymbol=&bindingp=0.5&position=CDS&targetscan=0&mirdb=0&mirtarbase=0&submit=set+filter**](http://mirwalk.umm.uni-heidelberg.de/interactions/?mirnaid=hsa-miR-650&genesymbol=&bindingp=0.5&position=CDS&targetscan=0&mirdb=0&mirtarbase=0&submit=set+filter)**.**


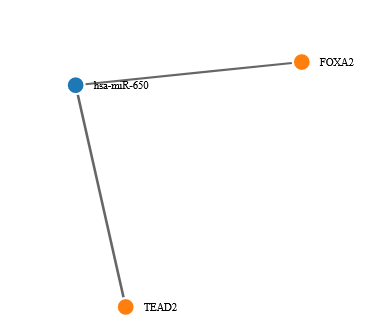

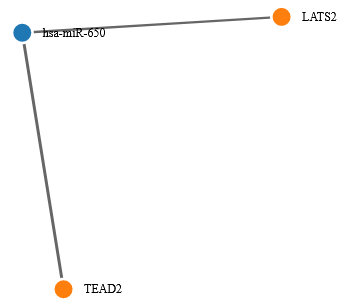


**Fig. S6C: Print screen showing the interaction between TEAD2 and LATS2 genes with miR-650 using** **miRWalk 3.0 available at** [**http://mirwalk.umm.uni-heidelberg.de/**](http://mirwalk.umm.uni-heidelberg.de/)**.**


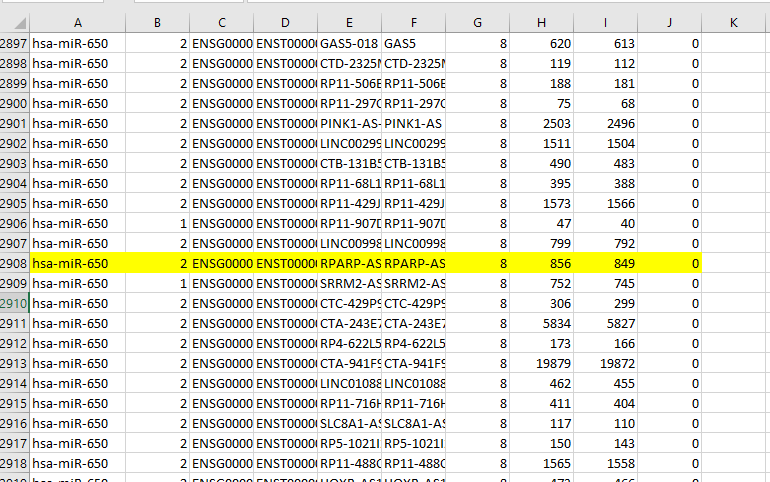


**Fig. S7A: Screen shot showing RPARP-AS1 LncRNA as a potential target using a mirwalk 2 available at** [**http://zmf.umm.uni-heidelberg.de/apps/zmf/mirwalk2/mir2ret/mir2mwlnc.php**](http://zmf.umm.uni-heidelberg.de/apps/zmf/mirwalk2/mir2ret/mir2mwlnc.php)**.**

**>hsa-mir-650 MI0003665**

**CAGUGCUGGGGUCUCAGGAGGCAGCGCUCUCAGGACGUCACCACCAUGGCCUGGGCUCUGCUCCUCCUCACCCUCCUCACUCAGGGCACAGGUGAU**

**Fig. S7B: Sequence of has-miR-650 using miRbase available at** [**http://www.mirbase.org/cgi-bin/get_seq.pl?acc=MI0003665**](http://www.mirbase.org/cgi-bin/get_seq.pl?acc=MI0003665)**.**

**> NONHSAT016119.2 ttttttttttttttttttttgtagagacagggtttcgccatgttgcctaggctggtctccaacctggtctcctgggctcaagcgatccgcccgcctcggcctcccacagtgctgggattccaggcgtgagctaccgcgcccggccTATTTACTTTTCTTACTAAGCTGGGGATCACCGTCGCCCTCGGCTTGGCAGGAAGGCGGGGGTGCAAGAAGAAAAGAGGTACAGAACACCCAGAGGTGCCCTCGATTCCGTCTTGCACTTGCCCTTCTCCCACCGTCCAGCAATAAAGCGAGAGAAACAAGTGCAGGAAACTGGCCGGCAGTCATGGGAGAAGCCAAAAAGACAGGTGAGCAGCGCGTCCGCCTCCCGCGCACAGCCTTGGGGAGCCGGCGGGATCCAGAGCGGGGCTCCTCTCCGCACTTTGTAGCTGCGTTGCTCCGCTCCATGCCCTGCCTCAGCCACTCCTCCTGGTTACCAGGCAAAAGGAAACACCTTGAGCTGGCCAGGAGCTACCAGCGTCTGTCTACACCTGGgctggagtgcagcggcatgatctcggcacactgcagccaccgtcttccaggttcaagcgattctcccacctcagcctcccgaggagctgggattacaggagttcagtggcatgaccagggctcactgcaaccttgatctgggctcaagtgatcctcctacctcagcttcctgagtagctaggaccacaggtgtgcaccaaccacacccgactaatttttgtagagatgagatcccactatgttacccaggctggtcttgaactcctgggctcaggtgatcatcctgccttggcttcccaaagtactgggattataggcttgagccaccgtgcctggccTGTGATCAGAATTCTCATTTTTTTAGTCACTAAaaatgctggggggcactccattctccattatgtgattagttcacattgcatgcttgtatcaaaacatcatatataccccacaaatatatacaAAAAACTTTAAAATTTTAAGTATTAATTGCTCAGGAAAAAATTAAAATGCTGGGGTGCTGAAATCTCAAGGGCCCCATTACAAAACTCCTTAGGAACCTCGCCCTCTCTCTGCTGTAAGGACTGGTTCCAGAATGAGAGAATTTAAAAGACATTCcccccccaaaatgttcataatgtcaccccggaaacctgcgaatatgttatattacatgaccagggagaagtaaggttgcagatggcagtaaggttgctaatgggctgaccttaagataaggagatgatcctggattatctgggtggacccaatgtaatcacaagggtccttaactgtggaatagtgaggtggctgagtcagaggcagagtgatgcaatgactgaaagacttaaccagccatcaccggctttgaatacggaagacggtcatgagccagggaatgcaggcaggctctgggagctgaaaaaagcaagaaaatggattctcccctggagcctccagaagggatgcggtcctgccaaccccttgtcagtgagccatttcagatttctgacttccaggactgtaagaaaataaacttgtcttgttttcagcca**

**Fig. S7C: Sequence of RPARP-AS1 LncRNA using noncode available at** [**http://www.noncode.org/show_rna.php?id=NONHSAT016119&version=2&utd=1#**](http://www.noncode.org/show_rna.php?id=NONHSAT016119&version=2&utd=1)


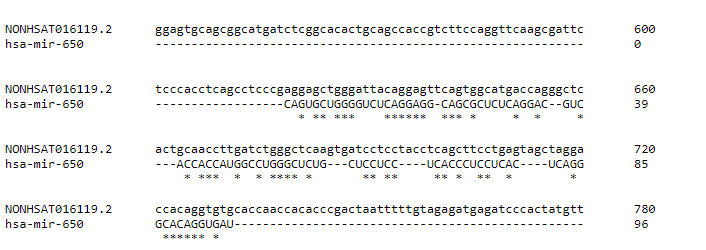
 **Fig. S7D: Screen shot showing alignment between hsa-mir-650 and RPARP-AS1 LncRNA**

**using Clustal Omegatool of ebi available at** [**https://www.ebi.ac.uk/Tools/services/web/toolresult.ebi?jobId=clustalo-I20210209-113823-0177-82478586-p1m&analysis=alignments**](https://www.ebi.ac.uk/Tools/services/web/toolresult.ebi?jobId=clustalo-I20210209-113823-0177-82478586-p1m&analysis=alignments)**.**
